# Identification of two QTLs associated with high fruit acidity in apple using pooled genome sequencing analysis

**DOI:** 10.1038/s41438-020-00393-y

**Published:** 2020-10-08

**Authors:** Seunghyun Ban, Kenong Xu

**Affiliations:** Horticulture Section, School of Integrative Plant Science, Cornell Agritech, Cornell University, Geneva, NY 14456 USA

**Keywords:** Plant genetics, Sequencing

## Abstract

Acidity is a critical component determining apple fruit quality. Previous studies reported two major acidity quantitative trait loci (QTLs) on linkage groups (LGs) 16 (*Ma*) and 8 (*Ma3*), respectively, and their homozygous genotypes *mama* and *ma3ma3* usually confer low titratable acidity (TA) (<3.0 mg ml^−1^) to apple fruit. However, apples of genotypes *Ma*- (*MaMa* and *Mama*) or *Ma3*- (*Ma3Ma3* and *Ma3ma3*) frequently show an acidity range spanning both regular (TA 3.0–10.0 mg ml^−1^) and high (TA > 10 mg ml^−1^) acidity levels. To date, the genetic control for high-acidity apples remains essentially unknown. In order to map QTLs associated with high acidity, two genomic DNA pools, one for high acidity and the other for regular acidity, were created in an interspecific F_1_ population Royal Gala (*Malus domestica*) × PI 613988 (*M. sieversii*) of 191 fruit-bearing progenies. By Illumina paired-end sequencing of the high and regular acidity pools, 1,261,640 single-nucleotide variants (SNVs) commonly present in both pools were detected. Using allele frequency directional difference and density (AFDDD) mapping approach, one region on chromosome 4 and another on chromosome 6 were identified to be putatively associated with high acidity, and were named *Ma6* and *Ma4*, respectively. Trait association analysis of DNA markers independently developed from the *Ma6* and *Ma4* regions confirmed the mapping of *Ma6* and *Ma4*. In the background of *MaMa*, 20.6% of acidity variation could be explained by *Ma6*, 28.5% by *Ma4,* and 50.7% by the combination of both. The effects of *Ma6* and *Ma4* in the background of *Mama* were also significant, but lower. These findings provide important genetic insight into high acidity in apple.

## Introduction

Apples are a favorite fruit worldwide and are consumed fresh or as processed products such as apple juice, contributing important nutrients to human health. Consumer preference in choosing apples is largely influenced by fruit acidity levels although other fruit quality attributes such as color, crispness, juiciness, and sugar content also play a considerable role^1^. Several organic acids are detectable in mature apples, including malic acid, citric acid, fumaric acid, succinic acid, maleic acid, and others^2^. However, malic acid is predominant and largely determines fruit acidity levels. Apples are classified into three groups according to titratable acidity (TA) and consumer acceptance: low (<3.0 mg ml^−1^ in malic acid equivalent), regular (3.0–10.0 mg ml^−1^), and high (>10.0 mg ml^−1^)^3,4^. Apples in low and high-acidity ranges usually are not suitable for fresh consumption. Due to its essential role in fruit quality, fruit acidity is routinely measured with TA to ensure fruit taste and flavor in apple breeding programs^5–7^. Meanwhile, fruit acidity also has been investigated intensively in apple genetics studies.

Early inheritance studies concluded that the low-acidity trait was governed by a single recessive gene *ma*^3,8,9^. The *Ma* locus had been genetically mapped to linkage group (LG) 16 in the apple genome^10^. This finding was confirmed in multiple studies using quantitative trait locus (QTL) mapping approach^4,11–13^. The *Ma* QTL has been characterized in detail, leading to identification of a strong candidate gene *Ma1* (MDP0000252114) encoding a protein closely related to the tonoplast localized aluminum-activated malate transporter (ALMT) 9 in *Arabidopsis*^14–16^. The genetic cause for low acidity had been attributed to the premature stop codon leading mutation from G to A at base 1455 in the *Ma1* open reading frame (ORF), which effectively truncates 84 amino acids at the MA1 C-terminus, presumably creating a malfunctioned MA1^14^. Indeed, a latest study demonstrates that the C-terminus of MA1 is essential for malate transport as the Ma1-1455A (allele *ma1*) protein showed significantly lower malate transport activity than the Ma1-1455G (*Ma1*) protein when expressed in both *Xenopus laevis* oocytes and *Nicotiana benthamiana* cells^17^.

Another important QTL for fruit acidity was identified on LG 8^12,13,18–21^, which was named *Ma3*^20^ following *Ma1* and *Ma2* (MDP0000244249), the two ALMT-like genes identified under QTL *Ma*^14^. Candidate genes for the *Ma3* QTL were proposed as well, such as MDP0000294924 encoding a ring finger and CHY zinc finger domain-containing protein^18^, MDP0000582174 encoding a MYB transcription factor and MDP0000239624 encoding a malic enzyme^19^, and MdPP2Ch (MDP0000141481) and MdSAUR37 (MDP0000153382)^22^. However, *Ma3* also has been shown to be less or none of a factor in other studies. For example, the QTL was detected only in one of the 2 years studied, and was regarded only one of the six minor QTLs for TA in the Telamon × Braeburn cross^11^. In addition to the major QTLs *Ma* and *Ma3*, minor fruit acidity QTLs were identified on LGs 2, 10, 13, 15, and 17^11^, and LGs 1 and 6^4^. These studies demonstrate that the genetic control of apple fruit acidity involves multiple loci with multiple alleles that can differ by genetic background of the germplasm considered.

In our effort to identify and characterize *Ma1* in population Royal Gala (*Malus domestica*) × PI 613988 (*M. sieversii*), we observed huge variation in fruit acidity in progenies of genotypes *MaMa* (4.1–18.7 mg ml^−1^) and *Mama* (3.2–14.1 mg ml^−1^) due to the presence of high-acidity progenies^4^. Interestingly, the effect of *Ma3* was undetectable in the population^4^, suggesting there are other unknown genes involved in high fruit acidity in the background of *Ma-* (*MaMa* and *Mama*). The objective of this study was to identify QTLs associated with high acidity in the background of *Ma-*.

## Materials and methods

### Plant materials

**Fig. 1:**
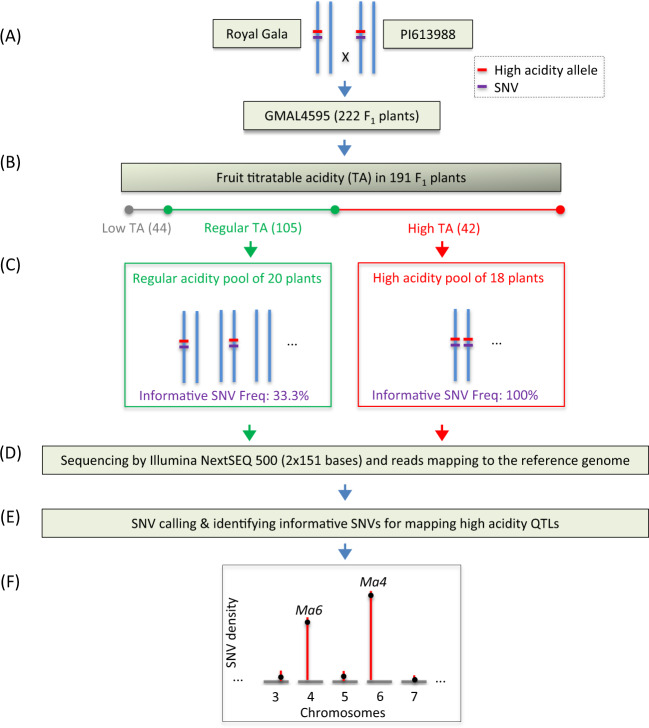
Schematic representation of the pooled genome-sequencing-based allele frequency directional difference and density (AFDDD) mapping of recessive high-acidity QTLs. **a** Creation of population GMAL 4595 (Royal Gala × PI 613988) of 222 F_1_ plants. The two parents were assumed to be heterozygous at the high-acidity locus. The vertical lines in blue represent homolog chromosomes. The red and purple short bars represent the high-acidity allele and SNVs in relation to the reference genome, respectively. **b** Evaluation of fruit titratable acidity (TA) in 191 F_1_ plants. The low (1.0–2.8 mg ml^−1^), regular (3.2–10.0 mg ml^−1^), and high (10.0–18.7 mg ml^−1^) acidity ranges were shown in gray, green, and red scales, respectively. The number of progenies in each TA range is given in parenthesis. **c** Creation of the high and regular acidity pools with 18 and 20 F_1_ segregants, respectively. The segregants were assumed to be homozygous at the high-acidity locus in the high-acidity pool while comprising three different genotypes (two heterozygotes and one homozygote without the high-acidity allele) in the regular acidity pool. As such, the expected informative SNV allele frequencies (AF) were 100% in the high-acidity pool and 33.3% in the regular acidity pool. **d** Sequencing the two genome pools and mapping the sequence reads against the apple reference genome^26^. **e** Calling out SNVs and identifying those informative for mapping, which were characterized with AF > 85% in the high-acidity pool and AFDD > 41.7 percentage points between high and regular acidity pools, lower than the expected difference of 51.7 (85–33.3%) percentage points to buffer variation. **f** AFDDD mapping of high-acidity QTLs by examining the genome-wide distributions of the informative SNVs. There were two highly significant SNV density peaks on chromosomes 4 and 6, designated *Ma6* and *Ma4*, respectively

The F_1_ population GMAL 4595 of 222 individuals and their associated fruit acidity data used in this study were obtained from an interspecific cross between Royal Gala (*Malus domestica*; *Mama*) and PI 613988 (*M. sieversii*; *Mama*) and were reported previously^4^. The seed parent Royal Gala is a widely grown cultivar, and the pollen parent is an elite selection of *M. sieversii* collected from Kazakhstan, which is a major progenitor species of domestic apples^23,24^. The fruit titratable acidity (TA) data from 191 of the 222 seedling trees were evaluated at harvest in 2010^4^ (Fig. 1a, b). Briefly, fruit were harvested when their Cornell Starch Index (CSI)^25^ were scored between CSIs 4 and 6. For each progeny, five to ten fruit were combined into a single pool and used for juice extraction. The measurement of juice TA was conducted using an autotitrator (Metrohm 848 Titrino Plus and Metrohm 869 Compact Sample Changer, Herisau, Switzerland), which showed a wide range of fruit acidity from 1.0 to 18.7 (mg ml^−1^) in malic acid equivalent. Based on the preference for fresh consumption of apples, fruit TA below, within and above the acceptable range (3–10 mg ml^−1^) were called low, regular, and high acidity, respectively.

### Genotyping at loci *Ma*, *Ma3*, and others

Genotyping at the *Ma* locus was conducted using a cleaved amplified polymorphic sequence (CAPS) marker CAPS1455 (Table S1)^14^. The associated restriction enzyme BspHI (New England Biolabs, Ipswich, MA, USA) directly detects the premature stop codon mutation from TGG_1455_ (allele *Ma1*) to TGA_1455_ (low-acidity allele *ma1*) at the 1455th base in the *Ma1* ORF^14^. Genotyping at the *Ma3* locus was carried out using the sequence-tagged site (STS) markers MdPP2CH and MdSAUR37 (Table S1) developed previously^22^, which were located at the 8.7th and 11.6th Mb on chromosome 8 in the GDDH13 apple reference genome^26^, respectively. Markers CAPS1455, MdPP2CH, and MdSAUR37 were analyzed using agarose (1.5%) gel electrophoresis. In addition, the genotypic data of simple sequence repeat (SSR) markers CH02g09 (in the *Ma3* region at the 10.0th Mb on Chromosome 8), C1902 (at the 1.5th Mb on Chr 17), and C14087 (at the 4.9th Mb on Chr 6) from a previous study^27^ were also used to help determine the effect of other putative acidity QTLs (Table S1).

### Construction and sequencing of two genomic DNA pools

Two genomic DNA pools, one for high acidity (12.6 ± 2.5 mg ml^−1^) and the other for regular acidity (5.7 ± 0.6 mg ml^−1^), were created using 18 and 20 progenies from the GMAL 4595 population, respectively (Table S2, Fig. 1c). Since *Ma3* was a non-factor in this population and the progenies in the high-acidity pool comprising five *MaMa* and 13 *Mama* progenies, similar to those in the regular acidity pool, i.e., 5 in *MaMa* and 15 in *Mama*, the acidity differences between the two pools were assumed to be caused by genetic factors other than *Ma* and *Ma3*. Genomic DNA samples were extracted from young leaves, and the DNA samples were assessed on 1% agarose gel for integrity, and were quantified with a Nanodrop 1000 spectrophotometer (Thermo Scientific, Wilmington, DE, USA). For each pool, an equal amount of DNA (1 µg) from each progeny was combined to produce the pooled genomes (Fig. 1c). Construction of the pooled genomic libraries was conducted using NEBNEXT Ultra DNA library prep kit for Illumina sequencing (New England Biolabs, E7370) with a targeted insert size of 500 bp. The libraries were sequenced in paired-end (2 × 151 bp) on an Illumina NextSeq 500 platform (Fig. 1d) at the Genomics Facility of Cornell University (Ithaca, NY, USA).

### Reads mapping against the apple reference genome and calling of DNA variants

The Illumina raw reads were trimmed and filtered to remove adaptors and low quality (0.01 as quality score p limit) reads. The cleaned reads from both pools were mapped to the apple reference genome GDDH13^26^ using software CLC Genomic Workbench (v7.5, CLC Bio, Cambridge, MA, USA) with the following parameters: similarity 0.98, length fraction 0.8, insertion cost 3, deletion cost 3, and mismatch cost 2. DNA variants from both pools were detected using fixed ploidy (diploid) variant detection tool available in CLC Genomic Workbench. Single-nucleotide variants (SNVs) that were associated with a read coverage range outside 20–200×, or with a complex genotype were all removed. Next, SNVs that were reference alleles were removed in order to avoid double counting in our downstream analyses. Only were the SNVs that passed these filters used for further analyses.

### Allele frequency directional difference and density (AFDDD) mapping

The AFDDD mapping approach^28^ was adapted in this study for mapping the high-acidity phenotype in the background of *Ma-* (*Ma* and *Mama*). Based on the approximate 3:1 (*p* = 0.3173) segregation between regular (105) and high (42) acidity in the *Ma-* progeny (Fig. 1b), the primary working hypothesis was that the high-acidity phenotype was controlled by one or few recessive genes and both parents were heterozygous at the gene loci. Alternative hypotheses were also considered to cover two possible cases: (1) the high-acidity phenotype was dominant over regular acidity but their segregation was distorted; and (2) the cross was *aabb* (Gala) × *AaBb* (PI 613988) or *Aabb* (Gala) × *aaBb* (PI 613988) and the high-acidity phenotype was controlled by two complementary genes in A-B-. With the high-acidity recessive hypothesis, the DNA variants of segregation type <**h**k > x <**h**k > would be most informative among the others^28^, where each letter represents one of the four DNA bases and the letters in bold represent variants in relation to the reference genome^26^. The alleles in each first and third positions are assumed linked in coupling phase to the recessive high-acidity allele in the two parents, respectively. We further inferred that the variants would be homozygous in the high-acidity pool, and heterozygous in the regular acidity pool. Considering the case involving a single recessive gene, the expected variant allele frequency (AF) in the high-acidity pool would be 100%, and that in the regular acidity pool would be 33.3% (Fig. 1c). Consequently, the expected variant allele frequency directional difference (AFDD) between the high and regular acidity pools would be 66.7 (100–33.3%) percentage points. In practice, we defined homozygous variants were those that were associated with AF > 85% and heterozygous variants with AF 15–85%, and informative variants were referred to those of AFDD > 41.7 percentage points between the high and regular acidity pools, a ten-percentage-point lower than the expected 51.7 (85–33.3%) percentage points to accommodate fluctuations (Fig. 1e). For mapping the high-acidity phenotype, the density of such informative DNA variants in 1-Mb moving windows was examined based on their distributions along each chromosome. Genomic regions of significantly higher SNV density than the genome average in the *z*-score tests were considered linked to the high-acidity phenotype (Fig. 1f). The cutoff was LODz (−log_10_P(z)) > 6.0.

### Marker development

High-resolution melting (HRM) and SSR markers were designed using program Primer3Plus^29^ to target SNVs and SSRs in the genomic regions (*Ma6* and *Ma4*) that were detected to be putatively associated with high acidity in AFDDD mapping (Table S1). The PCR and HRM analysis were performed using the CFX96-real time system (Bio-Rad Laboratories, Hercules, CA, USA) with Precision Melt Supermix (Bio-Rad Laboratories, Hercules, CA, USA) according to the manufacturer’s protocol. Briefly, 10 µl reactions with 20 ng of template DNA, 5 µl of Precision Melt Supermix, and 200 nM of forward and reverse primers were used. Two-step PCR and high-resolution melting analyses were carried out according to Precision Melt Supermix protocol. The thermo-cycling conditions included an initial DNA denaturation at 95 °C for 2 min, 40 cycles of 95 °C for 10 s, and 60 °C for 30, and a high-resolution melting analysis step comprising heteroduplex formation at 95 °C for 30 s and 60 °C for 1 min, and high-resolution melting and plate reading from 65 to 95 °C (heating rate 0.2 °C/10 s). Plate reads were incorporated after each PCR cycle and each melting step to record sample amplification and DNA melting information. Data were processed using the Bio-Rad CFX Maestro 1.0 and Bio-Rad Precision Melt Analysis 1.3 software packages. Analyses of SSR markers were performed as described previously^27^.

### Statistical analysis

One- or two-way analysis of variance (ANOVA) and Tukey’s honest significant difference (HSD) test were performed using R version 3.3.1^30^.

## Results

### Segregation of high acidity and associated markers

**Fig. 2:**
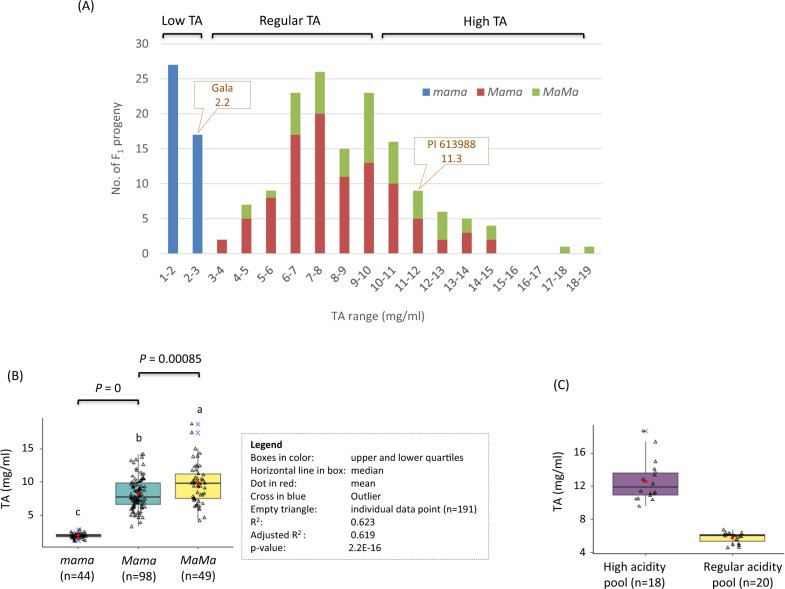
Analysis of the genetic effect of marker CAPS1455 (*Ma*) in population GMAL 4595. **a** Distribution of fruit acidity in the 191 F_1_ plants and their associated *Ma* genotypes. **b** ANOVA analysis of the genotypic effect of *Ma*. Alleles *Ma* and *ma* correspond to the relatively high and low acidity, respectively. **c** Fruit acidity levels of the F_1_ segregants used in the high and regular acidity pools

The high-acidity phenotype clearly segregated in population GMAL 4595. Among the 191 fruit-bearing progenies, 42, 105, and 44 were recorded with high (TA 10.0–18.7 mg ml^−1^), regular (3.2–10.0 mg ml^−1^), and low acidity (1.1–2.8 mg ml^−1^), indicating high fruit acidity was under the control of a few major genes. The segregation of the *Ma* (CAPS1455) alleles fit 1:2:1, i.e., 49 (*MaMa*):98 (*Mama*):44 (*mama*) in Chi-square test (*p* = 0.8217) (Fig. 2a, Fig. S1A). One-way ANOVA analysis of the genetic effect of *Ma* showed that *Ma* explained 61.9% (adjusted *R*^2^, *p* = 2.2E−16) of the fruit acidity variation in the population (Fig. 2b), confirming that *Ma* was a major genetic factor for apple fruit acidity in population GMAL 4595.

The effect of *Ma3* could not be detected when accessed using markers/genes MdPP2CH^22^, CH02g09^27^, and MdSAUR37^22^ that were located at the 8.7th, 10.0th, and 11.6th Mb in the *Ma3* region on chromosome 8 in the apple reference genome^26^. Based on how the MdPP2CH alleles were defined in relation to acidity (Fig. S1C)^22^, the genotypes of Gala and PI 613988 were homozygous low-acidity *ma3ma3 (AA)* and homozygous high-acidity *Ma3Ma3 (GG)*, respectively, and the genotype of their F_1_ progeny (GMAL 4595) was heterozygous *Ma3ma3 (AG)* (Fig. S1C), implicating a non-segregation population for *Ma3*. However, both MdSAUR37 and CH02g09 segregated in the population.

Marker MdSAUR37 detected the two alleles (bands) in the population as expected (Fig. S1B), corresponding to the high (*Ma3*) and low (*ma3*) acidity alleles, respectively^22^. Based on their allelic profiles, the genotype of Gala would be *Ma3ma3*, and that of PI 613988 would be *ma3ma3* (Fig. S1B). Therefore, non-parental genotypes would not be expected present in the population. However, the observed segregation was 50 (*Ma3Ma3*):39 (*Ma3ma3*):102 (*ma3ma3*). To account for the puzzling presence of the 50 non-parental genotype progenies, the pollen parent PI 613988 was assumed to carry a null allele of MdSAUR37, thereby having a *ma3-*null genotype. Under this hypothesis, the observed segregation would be 50 (*Ma3-null)*:39 (*Ma3ma3)*:102 (*ma3ma3* & *ma3-null*), fitting the expected segregation ratio 1:1:2 (*p* = 0.3410), seemingly in support of the null-allele hypothesis.

To resolve the compound genotype (*ma3ma3* & *ma3-null*) of MdSAUR37, SSR marker CH02g09 was used, which amplified three distinct alleles (bands) a, b, and c of approximate sizes 80-, 70-, and 65-bp, respectively, from the two parents, including alleles a and b from Gala and allele c from PI 613988 (Fig. S1D)^27^. However, allele c was present in only 54 of the 165 progenies of fruit that were genotyped^27^, indicating PI 613988 also carried a null allele (d) that could not be amplified (Fig. S1D), similar to what was inferred in the case of MdSAUR37. Since CH02g09 and MdSAUR37 were physically apart by ~1.6 Mb, and both showed a null allele in PI 613988, the c allele of CH02g09 was reasoned to be linked to the low acid allele (*ma3*) of MdSAUR37 in coupling phase. A genotype comparison between CH02g09 and MdSAUR37 suggested that alleles a and b in CH02g09 corresponded to the high (*Ma3*) and low (*ma3*) acidity alleles of MdSAUR37, respectively (Table S3). The segregation of the four CH02g09 alleles resulted in 25 (*Ma3ma3* = *ac*):55 (*Ma3-null* = *ad*):29 (*ma3ma3* = bc):56 (*ma3-null* = *bd*), indicating a significant distortion (*p* = 3.40E−4) from the expected 1:1:1:1 segregation (Table S3). ANOVA analyses demonstrated that MdSAUR37 and CH02g09 did not detect any significant effect of *Ma3* in any background of *Ma* (Figs. S2, S3). These analyses suggested that *Ma3* likely segregated as indicated by MdPP2CH in the population, i.e., Gala and PI 613988 had a homozygous low-acidity *ma3ma3* and homozygous high-acidity genotype *Ma3Ma3*, respectively. Therefore, the role of *Ma3* in fruit acidity variation was negligible among the F_1_ plants in the family.

A close look at the acidity distributions indicated that the segregation of low acidity was exclusively controlled by *Ma* as all 44 low-acidity progenies had the *mama* genotype (Fig. 2a). However, the segregation of high acidity could not be explained well by genotypes *MaMa* and *Mama* although fruit acidity was significantly higher (*p* = 8.5E−4) in *MaMa* (4.1–18.7 mg ml^−1^, *n* = 49) than *Mama* (3.2–14.1 mg ml^−1^, *n* = 98) (Fig. 2a, b). This was because the 42 high-acidity progenies comprised 20 in *MaMa* and 22 in *Mama*, suggesting that there be other fruit acidity genes in addition to *Ma* and *Ma3*. Since the segregation between regular acidity (105) and high acidity (42) fit 3:1 (*P* = 0.3173 in Chi-square test) in the progeny of genotypes *MaMa* and *Mama* (Fig. 2a), the high-acidity phenotype was reasoned to be recessive to regular acidity in the background of *Ma-*.

### AFDDD mapping of high acidity

To genetically map the recessive high-acidity phenotype, the high (12.6 ± 2.5 mg ml^−1^) and regular (5.7 ± 0.6 mg ml^−1^) acidity genomic DNA pools (Fig. 2c, Table S2) were sequenced and generated 163.2 and 228.0 million 2 × 151 bp paired-end raw reads (NCBI SRA accession PRJNA604459), respectively. Removing low quality reads, 157.3 million reads in the high-acidity pool and 224.9 million in the regular acidity pool were used for reads alignment onto the apple reference genome^26^, respectively (Table S4). As a result, 110.7 million reads (70.4%) in the high-acidity pool and 163.0 million reads (72.5%) in the regular acidity pool were mapped to the reference genome, equivalent to genome coverages 20.1× and 30.1×, respectively (Table S4). Using the variant detection tool available in the CLC Genomics Workbench, 1,261,640 SNVs that were present in both high and regular acidity pools were identified. Filtering these DNA variants that were not only homozygous in the high-acidity pool (AF > 85%), but also characterized with allele frequency directional difference (AFDD) > 41.7 percentage points between the high and regular acidity pools identified 1,017 SNVs. These SNVs were considered informative and useful in variant AFDDD mapping of the high-acidity phenotype under the working hypothesis that the trait was controlled by one or few recessive genes.

**Fig. 3:**
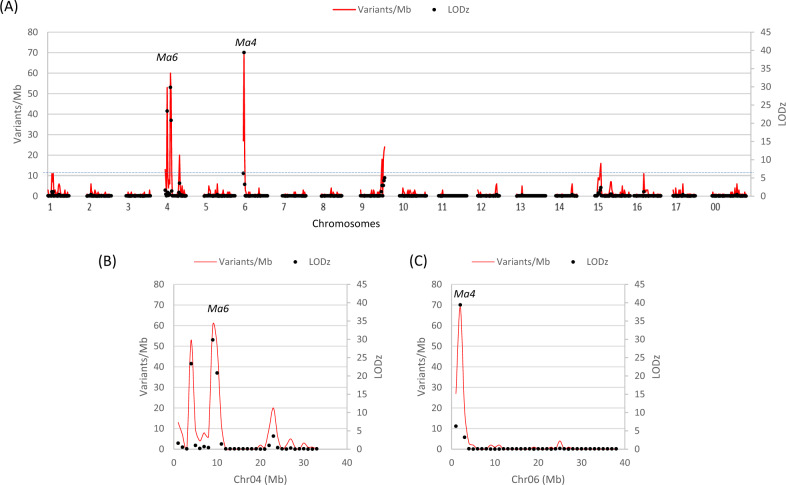
AFDDD mapping of high-acidity QTLs. **a** Genome-wide distribution of the 1017 informative SNVs for mapping high-acidity QTLs. The dashed line indicates the cutoff LODz (−log_10_P(z)) 6.0. The genomic regions under the two significant peaks on chromosomes 4 and 6 were dubbed *Ma6* (LODz = 29.9) and *Ma4* (LODz = 39.4) (**c**), respectively. **b**, **c** Close-up views of the informative SNV distribution on chromosomes 4 (**b**) and 6 (**c**), respectively

Examining the distribution of the 1017 SNVs in 1-Mb moving windows along the reference genome uncovered that there were two significant (LODz > 6.0) and highest peaks in variant density, designated *Ma6* and *Ma4* on chromosomes 4 and 6, respectively (Fig. 3a), indicating putative mapping of high acidity. The peak of *Ma6* (LODz = 29.9) was located in the 1 Mb region between the 8th and 9th Mb on chromosome 4 (Fig. 3b), whereas the peak of *Ma4* (LODz = 39.4) was positioned between the 1st and the 2nd Mb on chromosome 6 (Fig. 3c). The second highest peak on chromosome 4 was located between the 3rd and 4th Mb region (LODz = 23.4), physically close (5 Mb) to the *Ma6* peak, making it less likely an independent fruit acidity QTL from what was represented by *Ma6*.

### Confirmation of the mapping of *Ma6*

**Fig. 4:**
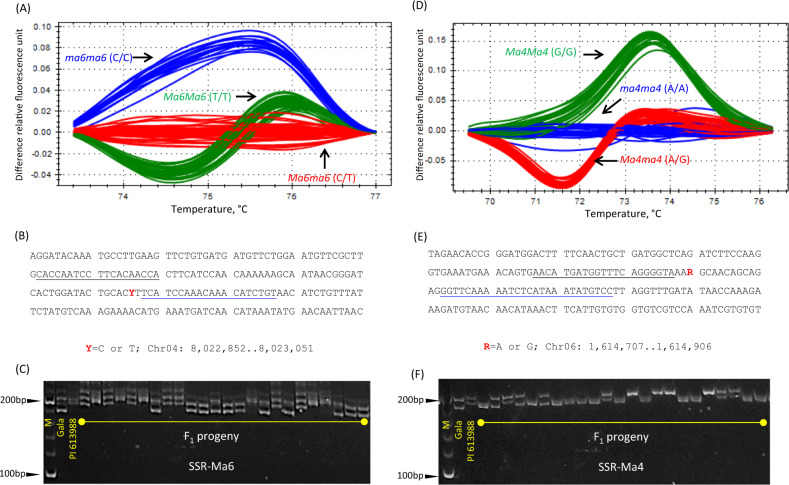
Development of HRM and SSR markers under QTLs *Ma6* and *Ma4* and their assays in population GMAL 4595. **a**, **d** Screen snapshot of high-resolution melting curves for markers HRM-Ma6 under *Ma6* (**a**) and HRM-Ma4 under *Ma4* (**d**). Each cluster of the melting curves (in red, green, and blue) represents one of the three genotypes at the targeting SNV site as indicated. **b**, **e** Flanking sequences of the SNV targeted by markers HRM-Ma6 (**b**) and HRM-Ma4 (**e**). Their chromosome coordinates were given and the targeting SNVs were shown red font with Y = T or C and R = A or G. The nucleotides corresponding to the HRM marker forward and reverse primers were underlined in black and blue, respectively. **c**, **f** Allelic profile of markers SSR-Ma6 (**c**) and SSR-Ma4 (**d**) as determined by 6% polyacrylamide gel electrophoresis (PAGE). Lane M stands for DNA ladders

**Fig. 5:**
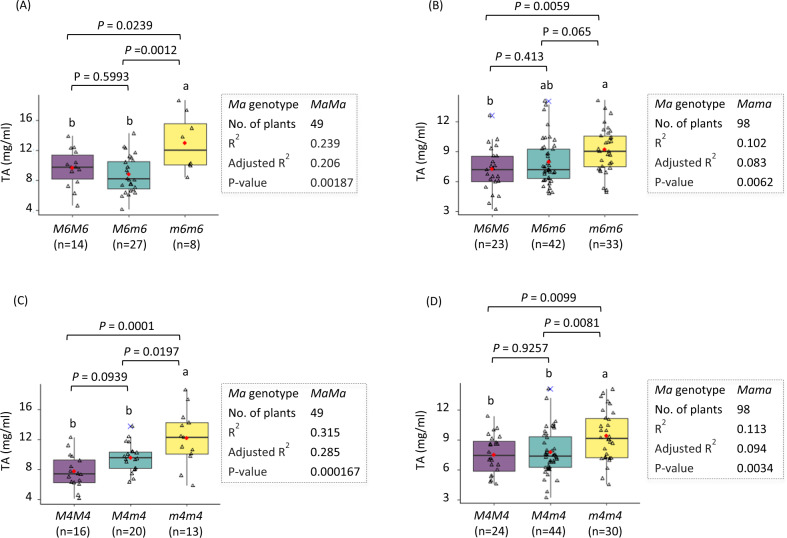
ANOVA analysis of the genetic effect of *HRM-Ma6* (**a**, **b**) and *HRM-Ma4* (**c**, **d**) on fruit acidity in the background of *MaMa* (**a**, **c**) and *Mama* (**b**, **d**). Alleles *Ma6*/*Ma4* and *ma6/ma4* correspond to the relatively low and high acidity, respectively. M6 = Ma6; M4 = Ma4. Different letters indicate significant difference (*p* < 0.05) between the genotype groups in Tukey’s HSD test. See also the legend in Fig. 1b

To confirm the mapping of *Ma6*, a high-resolution melting (HRM) marker targeting SNP T>C at Chr4_08,022,967, called HRM-Ma6, and an SSR marker located at 111 kb downstream of HRM-Ma6, named SSR-Ma6 were developed and used to genotype the entire population (Fig. 4a–c). HRM analysis of the marker revealed that there were three clusters of melting curves as expected, which correspond to genotypes *ma6ma6* (CC), *Ma6ma6* (CT), and *Ma6Ma6* (TT), respectively. The segregation of marker HRM-Ma6 was recorded with 54 *ma6ma6* (CC):84 *Ma6ma6* (CT):53 *Ma6Ma6* (TT), fitting the expected ratio 1:2:1 in Chi-square test (*p* = 0.2821), suggesting HRM-Ma6 segregated normally. ANOVA analysis of the effect of marker HRM-Ma6 demonstrated that it could account for 20.6% (*p* = 0.0019) of TA variation in the background of *MaMa* (*n* = 49) according to adjusted *R*^2^, and 8.3% (*p* = 0.0062) in the background of *Mama* (*n* = 98) (Fig. 5a, b), which were highly significant, thereby confirming the mapping of *Ma6* although its effect was not significant in *mama* (*n* = 44) and the entire population (Fig. S4A, B). In the background of *MaMa*, the progeny of genotype *ma6ma6* (CC) had significantly higher TA levels (12.9 ± 3.8 mg ml^−1^, *n* = 8) than those of genotypes *Ma6ma6* (CT) (8.8 ± 2.4 mg ml^−1^, *n* = 27, *p* = 0.0012) and *Ma6Ma6* (TT) (9.6 ± 2.6 mg ml^−1^, *n* = 14, *p* = 0.0239) (Fig. 5a) while the difference between genotypes *Ma6ma6* (CT) and *Ma6Ma6* (TT) was non-significant (*p* = 0.5993). A similar trend, despite a lessened degree, was also observed in the background of *Mama* (Fig. 5b). These results indicated that homozygous genotype *ma6ma6* (CC) confers high fruit acidity, supporting that a recessive gene under *Ma6* is relevant for high acidity. Marker SSR-Ma6 could detect all four possible distinct alleles of the two parents, segregating the progeny into four genotype groups (Fig. 4c). The estimated recombination frequency between HRM-Ma6 and SSR-Ma6 was 3.66% (14/382).

To test if the peak between the 3rd and 4th Mb on chromosome 4 represented an independent QTL, another HRM marker (HRM2-Ma6) was developed under the peak region (Fig. S5A, B). The recombination frequency between this marker and HRM-Ma6 was 8.64% (33/382). ANOVA analysis demonstrated that the effect of this marker was significant in both *MaMa* (adjusted *R*^2^ = 0.190; *p* = 0.0029) and *Mama* (adjusted *R*^2^ = 0.0595; *p* = 0.0202) (Fig. S5C, D), but to a lower degree if compared with the effect of HRM-Ma6 in the same background of *MaMa* (adjusted *R*^2^ = 0.206; *p* = 0.0019) and *Mama* (adjusted *R*^2^ = 0.083; *p* = 0.0062) (Fig. 5a, b), respectively. Moreover, the marker also did not show a significant effect in the background of *mama* and in the entire population (Fig. S5E, F), similar to HRM-Ma6. These observations suggested that the lower peak region was unlikely to represent a QTL independent from *Ma6*.

### Confirmation of the mapping of *Ma4*

Confirmation of the mapping of *Ma4* was conducted similarly as described above. In brief, another HRM marker, called HRM-Ma4, which detected SNP genotypes *ma4ma4* (AA), *Ma4ma4* (AG), and *Ma4Ma4* (GG) at base Chr6_1,614,796 was developed for estimating the effect of *Ma4* on high acidity in population GMAL 4595 (Fig. 4d, e). Based on Chi-square test, marker HRM-Ma4 also showed a normal 1:2:1 segregation (*p* = 0.2925) for the three genotypes: 50 *ma4ma4* (AA), 85 *Ma4ma4* (AG), and 56 *Ma4Ma4* (GG). In the background of *MaMa* (*n* = 49), the segregation of marker HRM-Ma4 explained 28.5% (adjusted *R*^2^; *p* = 1.67E−4) of the TA variation while 9.4% (*p* = 0.0034) in the background of *Mama* (*n* = 98), both of which were significant, confirming the mapping of *Ma4* (Fig. 5c, d). Interestingly, the effect of *Ma4* was also significant in *mama* (*n* = 44) and the entire population (Fig. S4C, D). A close look revealed a similar trend as observed for *Ma6*, i.e., the mean TA values of genotype *ma4ma4* (AA) were significantly higher than those values of genotypes *Ma4ma4* (AG) and *Ma4Ma4* (GG) in the background of both *MaMa* and *Mama* while the differences between genotypes *Ma4ma4* (AG) and *Ma4Ma4* (GG) were not significant (Fig. 5c, d), similarly supporting that a recessive gene under *Ma4* is critical for high acidity. An SSR marker positioned 120 kb upstream of HRM-Ma4, designated SSR-Ma4, was also developed, which was capable of detecting two distinct alleles from each of the two parents and segregating the progeny into four genotype groups (Fig. 4f). The recombination frequency between HRM-Ma4 and SSR-Ma4 was estimated 1.83% (7/382), lower than 3.66% calculated between HRM-Ma6 and SSR-Ma6 above.

### Genetic interactions between *Ma6* and *Ma4*

**Fig. 6:**
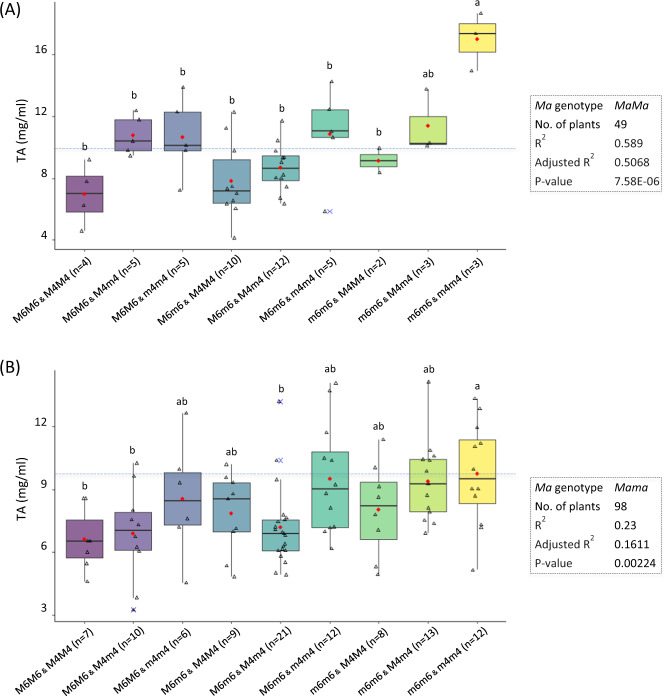
ANOVA analysis of the genetic effect of the interactions between *HRM-Ma6* and *HRM-Ma4* alleles on fruit acidity in the background of *MaMa* (**a**) and *Mama* (**b**). See also the legends in Figs. 1b and 5

A two-way ANOVA analysis based on the combined genotypes between markers HRM-Ma6 and HRM-Ma4 in population GMAL 4595 (Fig. 6) showed that the two markers explained 50.7% (adjusted *R*^2^, *p* = 7.58E−06) of the TA variation in the background of *MaMa* (*n* = 49), and 16.1% (*p* = 0.0022) in the background of *Mama* (*n* = 98), indicating that *Ma6* and *Ma4* had a strong additive effect on fruit acidity. The direct evidence in support of the large effect of *Ma6* and *Ma4* in the background of *MaMa* could be drawn from the following observations: a double homozygous recessive genotype *ma6ma6ma4ma4* was exclusively noted in the three progenies that had top three TA readings (15.0–18.7 mg ml^−1^) (Fig. 6a), whereas a double homozygous dominant genotype *Ma6Ma6Ma4Ma4* was associated with the four progenies that were within a TA range (4.6–9.2 mg ml^−1^) exclusively below 10 mg ml^−1^ (Fig. 6a). Notably, the expected frequency for these two homozygous genotypes in the background of *MaMa* was 0.0156 (0.25 × 0.25 × 0.25), equivalent to 2.98 out of the 191 progenies, close to what was observed for genotypes *MaMama6ma6ma4ma4* (3) and *MaMaMa6Ma6Ma4Ma4* (4). These results provided important genetic insight into how the interactions between *Ma6* and *Ma4* would determine fruit acidity and why ultrahigh and regular acidity progeny were co-present in the background of *Ma-*.

### Genes annotated in the *Ma6* and *Ma4* regions

In the 1 (8th to 9th)-Mb peak region of *Ma6* on chromosome 4 (Fig. 3b) and the 1 (1st to 2nd)-Mb peak region of *Ma4* on chromosome 6 (Fig. 3c), 61 and 73 genes (Tables S5 and S6) were annotated in the apple reference genome^26^, respectively. As a preliminary step toward the identification of candidate genes, the already annotated genes were also annotated in MAPMAN^31^ terms to better understand their potential functions. The 61 genes under the peak region of *Ma6* included 56 protein-encoding genes (the other five encode rRNAs and tRNAs), of which 48 were annotated with a putative function while the remainder eight were not. Below are 16 of the 48 genes deemed of various regulatory roles in important biological process, including (1) four transcription factors (MD04G1061200-homeobox protein 2, MD04G1063600-a C2H2-like zinc finger protein, MD04G1064500-a myb domain protein 3r-4, and MD04G1066800-a GRAS family transcription factor); (2) two transcription regulators (MD04G1061900-a CCHC-type zinc finger and MD04G1062700-a methyltransferase MT-A70 family protein); (3) two signal transducers (MD04G1065600-a IQ-domain 22, and MD04G1066000-a PAS domain-containing protein tyrosine kinase family protein); (4) four protein posttranslational modifiers (MD06G1014800, MD06G1009600, and MD06G1009700, which all encode a protein kinase superfamily protein, and MD06G1013000 for a RING/U-box superfamily protein); and (5) four protein degradation proteins (MD04G1064900-a C3HC4 type-ring finger family protein, MD04G1060900-a ubiquitin-protein ligases, MD04G1065500-a RHOMBOID-like 2, and MD04G1063000-a matrixin family protein).

In the *Ma4* peak region, the 73 annotated genes included one (MD06G1011800) encoding a miRNA (miR168), 56 encoding a protein of putative function, and 16 others without an annotation (13) or encoding an rRNA (3). Among the 56 genes of a putative function, ten genes were related to transcription regulation, including four encoding a MADS-box transcription factor (MD06G1013100, MD06G1013200, MD06G1013500, and MD06G1013600), two encoding a plant regulator RWP-RK family protein (MD06G1009200 and MD06G1009300), and MD06G1010000-a DNA-binding bromodomain-containing protein, MD06G1012500-a transcription factor Jumanji domain-containing protein, MD06G1014300-a Mob1/phocein family protein, and MD06G1014700-a growth-regulating factor 5. The other genes of interest included three encoding a protein kinase superfamily protein of roles in posttranslational protein modification (MD06G1014800, MD06G1009600, and MD06G1009700), MD06G1012300 encoding a signaling protein (RHO guanyl-nucleotide exchange factor 14), and MD06G1013000 encoding a RING/U-box superfamily protein involved in protein degradation.

### Investigation of alternative hypothesis in genetic control of high acidity

Two hypotheses not considering high-acidity recessive to regular acidity were tested to see how the high-acidity phenotype would be mapped by AFFDD mapping. The first hypothesis was that the high-acidity phenotype was dominant over regular acidity, but the segregation was distorted to 3:1 as observed (Figs. 1b and 2a). Under this scenario, the strategy for mapping a dominant phenotype^28^ was directly employed by identifying the most commonly used type of informative SNVs (type I), which were characterized by being specific to the high-acidity pool and by an expected SNV allele frequency of 50% or a range of 40–60%. Consequently, 8277 such SNVs were identified and were plotted against the apple reference genome (Fig. S6A). It showed that the most significant SNV density peak was reported between the 1st and 2nd Mb on chromosome 17 although a significant peak was also detected at the 3rd to 4th Mb on chromosome 4 (same as the second peak in the *Ma6* region) and at the 1st to 2nd Mb on chromosome 6 (same location as *Ma4*) (Fig. S6B-D). However, the putative mapping by the highest peak on chromosome 17 could not be validated in *MaMa* (*p* = 0.1450, *n* = 46) and in *Mama* (*p* = 0.1084, *n* = 85) based on ANOVA analysis of an SSR marker C1902 (Table S1, Fig. S6E, F) that was polymorphic for both Gala and PI 613988^27^, and was located at the 1.5th Mb within the peak region on chromosome 17^26^. These results demonstrated that the hypothesis led to a false positive mapping of a locus with the most significant *p*-value in the test, although the *Ma6* and *Ma4* regions were also detected.

The second hypothesis was that the high-acidity phenotype required two complementary genes and the progeny was derived from a cross of *aabb* (Gala) × *AaBb* (PI 613988) or *Aabb* (Gala) × *aaBb* (PI 613988). In either case, the progeny would segregate into 3:1 between regular acidity (1 *Aabb*, 1*aaBb*, and 1 *aabb*) and high acidity (1 *AaBb*), the same as what was observed (Figs. 1b and 2a). Further, the expected frequency of SNVs linked to A or B would be 50% in the high-acidity pool and 16.7% (1/6) in the regular acidity pool. Based on such expected SNV frequencies, we selected SNVs that had allele frequencies ranging from 40 to 60% in the high acid pool and from 6.7 to 26.7% in the regular acid pool, and that they differed by at least 23.3 percentage points, ten-percentage points below the expected 33.3 (50–16.7%) to accommodate variation. Applying these filters identified 6595 SNVs informative for AFDDD mapping, detecting a single significant peak between the 4th and 5th Mb on chromosome 6 (Fig. S7A, B), close to the peak of *Ma4* (between the 1st and 2nd Mb). However, confirmation of the mapping of this peak region with SSR marker C14087 (Table S1, Fig. S7C, D), which segregated for the PI 613988 alleles^27^ and located at the 4.9th Mb on chromosome 6^26^, showed that the marker effect was non-significant in *MaMa* (*p* = 0.0804, *n* = 44) while significant in *Mama* (*p* = 0.0466, *n* = 83), much lower than the effect of *Ma4* (Fig. 5c, d). Moreover, the *Ma6* region was never detected under this hypothesis.

## Discussion

### Population characteristics in GMAL 4595 and the AFDDD mapping approach

This study reported the identification of two recessive gene loci *Ma6* and *Ma4* that largely determine high acidity in apple in the background of *MaMa* as well as *Mama*. The success could be attributed to the choice of the mapping population GMAL 4595 and the adaptation to the AFDDD mapping approach. Population GMAL 4595 was previously used for fine genetic mapping of *Ma*, leading to the identification of the critical premature stop codon mutation in *Ma1* that was causal for low acidity^4,14,17^. One of the important characteristics of population GMAL 4595 was the non-detectable effect of *Ma3* on chromosome 8, making it highly suitable for detection of other genes controlling high acidity in the background of *Ma-*. The non-detectable effect of *Ma3* was likely caused by a homozygous low-acidity genotype *ma3ma3* in the seed parent Gala and a homozygous high-acidity genotype *Ma3Ma3* in the pollen parent PI 613988 as they would produce a non-segregation genotype *Ma3ma3* in the mapping population GMAL 4595. The three markers/genes studied in the *Ma3* region were MdPP2CH^22^, CH02g09^27^, and MdSAUR37^22^, which were located at the 8.7th, 10.0th, and 11.6th Mb on chromosome 8 in the apple reference genome^26^, respectively. Based on the published allelic profile of marker MdPP2CH^22^, the genotypes of Gala, PI 613988, and their F_1_ progeny were inferred to be *ma3ma3 (AA), Ma3Ma3 (GG)*, and *Ma3ma3 (AG)*, respectively (Fig. S1C), directly in support of the notion that the non-detectable effect of *Ma3* was caused by an identical genotype (*Ma3ma3*) in population GMAL 4595. In addition, no significant effect on TA was detected for the MdSAUR37 and CH02g09 alleles segregated in both parents and the population (Figs. S2 and S3), which indirectly supported that the genes underlying *Ma3* did not segregate in GMAL 4595. Notably, both genes *MdPP2CH* and *MdSAUR37* were implicated to be associated with malic acid accumulation in apple fruit^22^. The non-detectable effect by MdSAUR37 contradicted to its supposed role in fruit acidity in their study. This was because the inability to separate the low-acidity genotype *ma3ma3*, a signature genotype–phenotype association for *Ma3*, from the compounded genotype group of *ma3ma3* & *ma3-null* in MdSAUR37 (Fig. S2, Table S3) was largely resolved in CH02g09 and still CH02g09 showed a non-significant effect on acidity (Fig. S3, Table S3). Another useful character of GMAL 4595 was that the two parents were heterozygous at both loci *Ma6* and *Ma4*. Such type of segregation maximized the frequency of *ma6ma6* and *ma4ma4* and their effect on high acidity, which helped explain the wide acidity ranges in the background of *Ma-*.

The AFDDD mapping approach was initially developed for mapping a dominant trait (weeping) in apple based on pooled genome-sequencing analysis^28^. A major advantage of AFDDD mapping approach over the conventional QTL mapping approach in apple is its capacity for mapping without the need of construction of a genome linkage map and its effectiveness in identification of informative SNVs in the causal gene regions from a large pool of DNA variants that are usually under the control various segregation types due to its highly heterozygous genome^28^. In this study, the AFDDD mapping approach was adapted successfully in mapping high-acidity QTLs *Ma6* and *Ma4* in the background of *Ma-*, demonstrating its utility in detecting QTLs for recessive phenotype in apple, a self-incompatible heterozygous woody species, even when a major gene was involved. In self-compatible crops, the power of the pooled genome-sequencing-based approaches has been documented not only in QTL or mutation mapping, such as rice^32^, wheat^33^, and ryegrass^34^, but also in direct identification of causal genes or mutations, such as tomato^35^, lettuce^36^, peach^37,38^, and others.

### Effect of *Ma6* and *Ma4* on fruit acidity and marker-assisted selection (MAS)

The presence of low-acidity seedlings in apple breeding populations could be explained well with the major QTLs *Ma*^4^ and *Ma3*^22^. However, the genetic factors associated with the segregation of (ultra) high-acidity seedlings have been largely unknown. The identification of *Ma6* and *Ma4* by this study shed light on the genetic control of high acidity in the background of *MaMa* and *Mama* in apple. The estimated genetic effect of *Ma6* was 20.6% in *MaMa* and 8.3% in *Mama*, slightly lower than those of *Ma4*, which were 28.5% and 9.4%, respectively. Notably, the effect of *Ma6* and *Ma4* was much lower in the background of *Mama* than in that of *MaM*a. A most likely factor contributing to the observation is the documented highly unequal effect (on TA) of the *Ma* alleles between Gala (42.3%) and PI 613988 (17.0%)^4^. The other possible factors may include genetic recombinations between the markers used and the causal genes underlying *Ma6* or *Ma4*, and random experimental errors in TA data collections, which was conducted in 1 year. *Ma6* was a novel QTL relevant for fruit acidity as fruit acidity QTLs have not been reported on chromosome 4. However, *Ma4* likely represented the minor QTL previously detected on chromosome 6 near marker C12360^4^ as the marker is physically located at the 0.576th Mb, close to the peak of *Ma4* (1–2 Mb) on chromosome 6 in the reference genome^26^. The detection of a larger effect of *Ma4* in this study was probably benefited from the fact that HRM-Ma4 was an informative marker for both parents while C12360 was informative for Royal Gala only^4,27^. Another difference between the two QTLs was that *Ma6* showed a non-detectable effect in *mama* (Fig. S4A) while *Ma4* was also a significant factor (Fig. S4C). One possible explanation would be that the function of *Ma6* might require the presence of *Ma*, while that of *Ma4* could be independent of *Ma*.

The combined genetic effect of *Ma6* and *Ma4* was high (50.7%) in the background of *MaMa* while moderate (16.1%) in *Mama* (Fig. 6), suggesting that the associated markers HRM-Ma6/SSR-Ma6 and HRM-Ma4/SSR-Ma4 would be useful for removal of high-acidity seedlings that have a *MaMa* genotype. Based on the TA readings in the background of *MaMa*, all the three seedling trees of genotype *ma6ma6ma4ma4* were shown with ultrahigh acidity (15.0–18.7 mg ml^−1^) (Fig. 6a), indicating that such double homozygous high acid progenies could be safely discarded without risk. Remarkably, the observed frequency for the ultrahigh acidity genotype *MaMama6ma6ma4ma4* was 0.0157 (3/191), nearly equal to what was expected, i.e., 0.0156 (0.25 × 0.25 × 0.25). It is probably also a low-risk practice to rid of seedlings of genotypes (*ma6ma6Ma4ma4*; 10.1–13.8 mg ml^−1^, *n* = 3) and *Ma6ma6ma4ma4* (10.6–14.3 mg ml^−1^ in four of the five progenies while the remainder was of TA 5.9 mg ml^−1^), which are homozygous high-acidity genotype at one locus while heterozygous at the other. In theory, these three genotype groups may account for 7.8% (5/64) of the entire F_1_ population derived from a typical cross involving two heterozygous parents, i.e., *MamaMa6ma6Ma4ma4* × *MamaMa6ma6Ma4ma4*. Therefore, combining markers CAPS1455^14^, HRM-Ma6/SSR-Ma6, and HRM-Ma4/SSR-Ma4 could increase breading efficiency by 32.8% (21/64), of which 25.0% was contributed by the low acid (*mama*) seedlings and 7.8% by the high acid seedlings. These markers could also be used in planning of new crosses to minimize the frequency of the three high-acidity genotypes (*ma6ma6ma4ma4*, *ma6ma6Ma4ma4*, and *Ma6ma6ma4ma4*). One possible approach would be to choose one parent of homozygous genotype *Ma6Ma6* and the other parent of *Ma4Ma4*. However, more studies are needed to test if and how the *Ma6* and *Ma4* associated markers could be used in diverse apple breeding populations in which *Ma3* is or is not a factor. After all, the identification of *Ma6* and *Ma4* by this study was accomplished using a single interspecific cross (GMAL 4595) in which all progenies had the same genotype *Ma3ma3*.

### Alternative genetic models considered for mapping high acidity

The mapping of QTLs *Ma6* and *Ma4* was accomplished under the hypothesis that the high-acidity phenotype was recessive to regular acidity, which was a straightforward interpretation of the observed 3:1 ratio (*p* = 0.3173) between the regular acidity progeny (105) and the high-acidity progeny (42) in genotypes *MaMa* and *Mama* (Figs. 1b and 2a). However, such segregation could also be possible under the two hypotheses: (1) the high-acidity phenotype was dominant over regular acidity, but the normal segregation was distorted; (2) the cross was *aabb* (Gala) × *AaBb* (PI 613988) or *Aabb* (Gala) × *aaBb* (PI 613988) and the complementary interactions between genes (alleles) A and B were required for high acidity. AFDDD mapping analyses under the two hypotheses demonstrated that the results were less desirable, if not unacceptable, due to either a false positive mapping of the supposedly most significant peak on chromosome 17 under the first hypothesis (Fig. S6), or the complete miss of *Ma6* under the second hypothesis (Fig. S7), indirectly endorsing the high-acidity recessive hypothesis, under which the *Ma6* and *Ma4* QTLs were identified.

### Candidate genes under *Ma6* and *Ma4*

There were 61 genes annotated under the 1-Mb peak region of *Ma6* and 48 of them were indicated with a putative function (Table S5). Similarly, in the 1-Mb peak region of *Ma4*, 73 genes annotated, of which a miR168 encoding gene and 56 protein-encoding genes were given a putative function (Table S6). Although it would be difficult, if not impossible, to confidently suggest any candidate genes due to the large number of genes involved, the listed genes provided an important starting point to search for the candidate causal genes underpinning QTLs *Ma6* and *Ma4* that were associated high acidity in apple. Certainly, a much more dedicated study is needed to accomplish this goal in the future.

In conclusion, using pooled genome-sequencing-based AFDDD mapping approach, we identified two QTLs associated with recessive high-acidity phenotype in the background of *MaMa* and *Mama* in apple, which were localized on chromosomes 4 (*Ma6*) and 6 (*Ma4*), respectively. Their recessive genotypes *ma6ma6* and *ma4ma4* confer high acidity, which is in contrast with the recessive genotypes *mama* and *ma3ma3* that control low acidity. These findings not only provide important insight into genetic control of high acidity in apple, but also help enhance MAS by parental line selection and by removal of (ultra) high-acidity seedlings while low-acidity seedlings can be discarded according to *mama*. The genes annotated under *Ma6* and *Ma4* offer a list of candidates that might be genetically causal for high acidity.

## Additional notes

The two QTLs on chromosomes 4 and 6 had been named *Ma4* and *Ma5*, respectively, until the production proofreading stage of this manuscript. However, the same names (*Ma4* and *Ma5*) were also used in a relevant article (ref. ^39^) that was just published online on 8 September 2020. To avoid confusions, the original name *Ma4* on chromosome 4 was simply changed to *Ma6* and that on chromosome 6 to *Ma4*, and so *Ma6* appears before *Ma4* throughout this manuscript.

## Supplementary information

Supplementary information
