# Supplementary material for: Identification of two QTLs associated with high fruit acidity in apple using pooled genome sequencing analysis
Source: Hortic Res. 2020 Oct 8;7:171. doi: 10.1038/s41438-020-00393-y (PMC7546611; doi:10.1038/s41438-020-00393-y)
Supplement: Supplementary file 1 — Supplementary information [file 41438_2020_393_MOESM1_ESM.pdf]

Identification of two QTLs associated with high fruit acidity in apple using pooled genome sequencing analysis

Seunghyun Ban[1] and Kenong Xu[1*]

[1]Horticulture Section, School of Integrative Plant Science, Cornell Agritech, Cornell University, Geneva, NY 14456, USA.

*Correspondence:

Kenong Xu

kx27@cornell.edu

Supplementary information

- **Fig. S1.** Gel profile of DNA markers in the *Ma* and *Ma3* regions.
- **Fig. S2.** ANOVA analysis of the genetic effect of marker MdSAUR37 (*Ma3*) on fruit acidity in population GMAL 4595.
- **Fig. S3.** ANOVA analysis of the genetic effect of marker CH02g09 (*Ma3*) on fruit acidity in population GMAL 4595.
- **Fig. S4.** ANOVA analysis of the genetic effect of *Ma6* and *Ma4* on fruit acidity in the background of *mama* and in all 191 F_1 plants.
- **Fig. S5.** Development of marker HRM2-Ma6.
- **Fig. S6.** AFDDD mapping of high acidity as a dominant phenotype.
- **Fig. S7.** AFDDD mapping of high acidity under the control of two complementary genes.
- **Table S1.** Primer sequences of DNA markers
- **Table S2.** F_1 segregants used for pooling of genomes and their acidity and genotypes at loci *Ma (CAPS1455)* and *Ma3 (MdPP2CH* and *MdSAUR37)*
- **Table S3.** Comparison in the progeny of GMAL 4595 that were genotyped by both MdSAUR37 and CH02g09
- **Table S4.** Summary of reads mapping in pooled genome sequencing analysis
- **Table S5.** List of all genes annotated under the peak of QTL *Ma6* in the apple reference genome [27].
- **Table S6.** List of all genes annotated under the peak of QTL *Ma4* in the apple reference genome [27]

Fig. S1. Gel profile of DNA markers in the *Ma* and *Ma3* regions. Arrows in black show the size (bp) of DNA ladders in lanes marked with letter M. (A-C) Agarose gel profile of markers CAPS1455-*Ma* (A), MdSAUR37-Ma3 (B) and MdPP2CH-Ma3(C). Their relatively high (*Ma* or *Ma3*) and low (*ma* or *ma3*) acidity alleles were indicated by arrows in red. (D) Allelic profile of SSR marker CH02g09 as determined by 6% polyacrylamide gel electrophoresis (PAGE). As indicated, CH02g09 amplified three distinct alleles (bands) a, b, and c of approximate sizes 80-, 70-, and 65-bp, respectively, from the two parents, including alleles a and b from Gala and allele c from PI 613988.

Fig. S2. ANOVA analysis of the genetic effect of marker MdSAUR37 (*Ma3*) on fruit acidity in population GMAL 4595 in the background of *MaMa* (A), Mama (B), mama (C), and in all 191 F_1 progenies (D). *Ma3* and *ma3* stand for high and low acidity alleles of the marker, respectively. 'Null' signifies null allele in PI 613988. See also the legend in Fig. 1B.

Fig. S3. ANOVA analysis of the genetic effect of marker CH02g09 (*Ma3*) on fruit acidity in population GMAL 4595 in the background of *MaMa* (A), Mama (B), mama (C), and in all 165 F_1 progenies (D). *Ma3* and *ma3* stand for high and low acidity alleles of the marker, respectively. 'Null' signifies null allele in PI 613988. Same letters indicate non-significant difference (p>0.05) between the genotype groups in Tukey's HSD test. See also the legend in Fig. 1B.

Fig. S4. ANOVA analysis of the genetic effect of *Ma6* (A, B) and *Ma4* (C, D) on fruit acidity under the background of *mama* (A, C) and in all 191 F_1 plants (B, D). Alleles *Ma6*/*Ma4* and *ma6/ma4* correspond to the relatively low and high acidity, respectively. M6=Ma6; M4=Ma4. Different letters indicate significant difference (p<0.05) between the genotype groups in Tukey's HSD test.

Fig. S5. Development of marker HRM2-Ma6 under the second peak in the *Ma6* region and its assays in population GMAL 4595. (A) Screen snapshot of high-resolution melting curves for the marker. Each cluster of the melting curves (in red, green and blue) represents one of the three genotypes at the targeting SNV site as indicated. (B) Flanking sequences of the SNV targeted by the marker. Their chromosome coordinates were given and the targeting SNV was shown red font with Y=T or C. The nucleotides corresponding to the HRM marker forward and reverse primers were underlined in black and blue, respectively. (C-F) ANOVA analysis of the genetic effect of HRM2-Ma6 on fruit acidity in GMAL4595. See also the legend in Fig. 1B.

Fig. S6. AFDDD mapping of high acidity as a dominant phenotype. (A) Genome-wide distribution of the 8,227 informative SNVs for mapping high acidity QTLs. The dashed-line indicates the cutoff of LODz (-$\log_{10}P(z)) > 6.0$. The genomic regions under the three significant peaks were located on chromosomes 4, 6 and 17, respectively. (B)-(C) Close-up views of the informative SNV distribution on chromosomes 4 (B), 6 (C) and 17 (D), respectively. (E-F) ANOVA analysis of the genetic effect of an SSR marker C1902 under the peak region on chromosome 17 on fruit acidity in the background of *MaMa* (E) and *Mama* (F). Alleles a and b were from Gala, c and d from PI 613988. Same letters indicate non-significant difference ($p>0.05$) between the genotype groups in Tukey's HSD test. See also the legend in Fig. 1B.

Fig. S7. AFDDD mapping of high acidity under the control of two complementary genes. (A) Genome-wide distribution of the 6,595 informative SNVs for mapping high acidity QTLs. The dashed-line indicates the cutoff of LODz (-$\log_{10}P(z)) > 6.0$. The genomic region under a significant peak was located on chromosome 6. (B) Close-up view of the informative SNV distribution on chromosome 6. (C-D) ANOVA analysis of the genetic effect of an SSR marker C14087 under the peak region on chromosome 6 on fruit acidity in the background of *MaMa* (C) and *Mama* (D). Alleles c and d were from PI 613988. Same letters indicate non-significant difference ($p>0.05$) between the genotype groups in Tukey's HSD test. See also the legend in Fig. 1B.

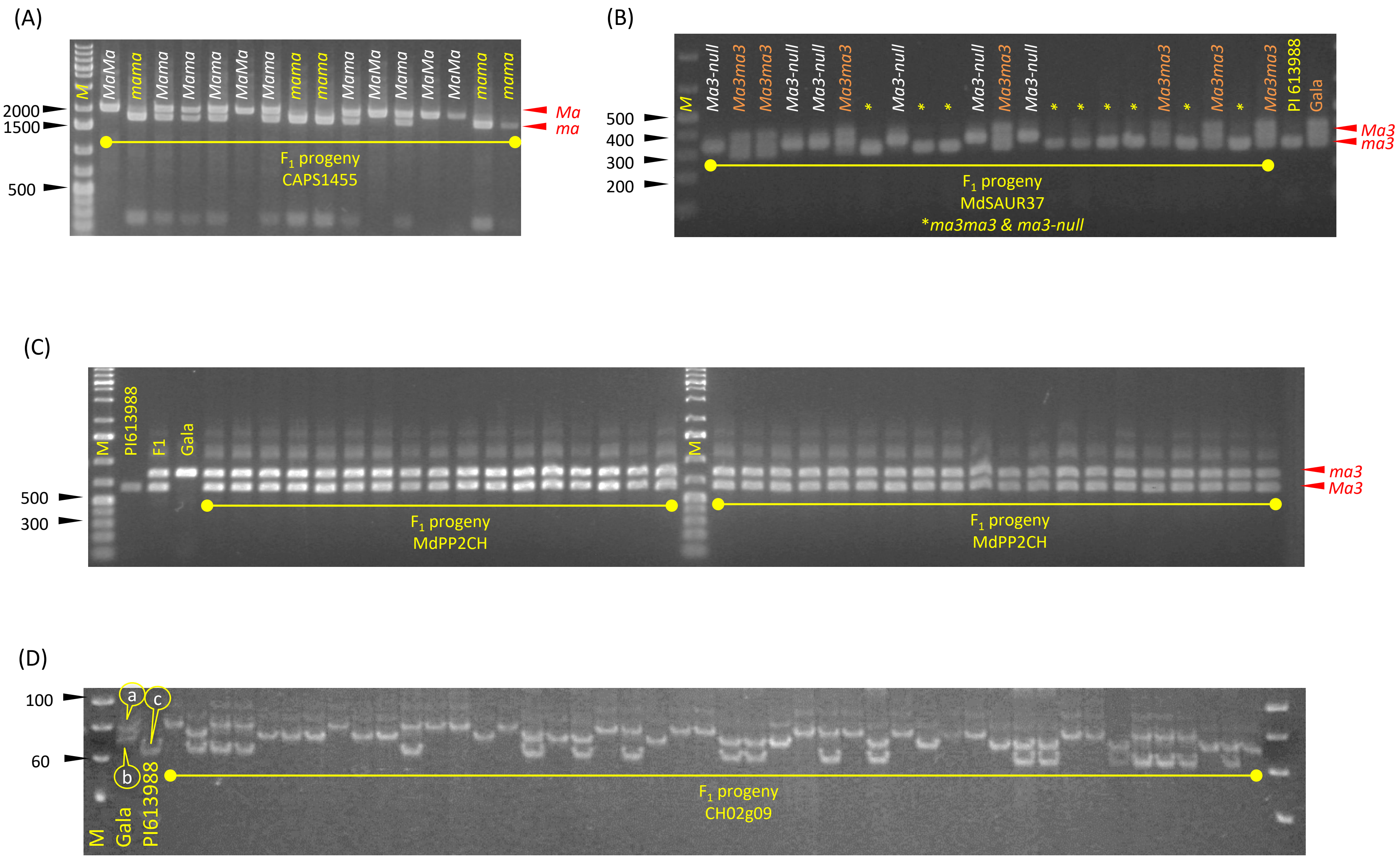
(A)
M
MaMa
mama
Mama
Mama
Mama
MaMa
Mama
mama
mama
Mama
MaMa
Mama
MaMa
MaMa
mama
mama
2000
1500
500
Ma
ma
F1 progeny
CAPS1455
(B)
M
Ma3-null
Ma3ma3
Ma3ma3
Ma3-null
Ma3-null
Ma3ma3
Ma3-null
Ma3-null
Ma3ma3
Ma3-null
Ma3ma3
Ma3ma3
Ma3ma3
PI 613988
Gala
500
400
300
200
Ma3
ma3
F1 progeny
MdSAUR37
*ma3ma3 & ma3-null
(C)
M
PI613988
F1
Gala
500
300
ma3
Ma3
F1 progeny
MdPP2CH
F1 progeny
MdPP2CH
(D)
100
60
a
b
c
M
Gala
PI613988
F1 progeny
CH02g09

Fig. S1

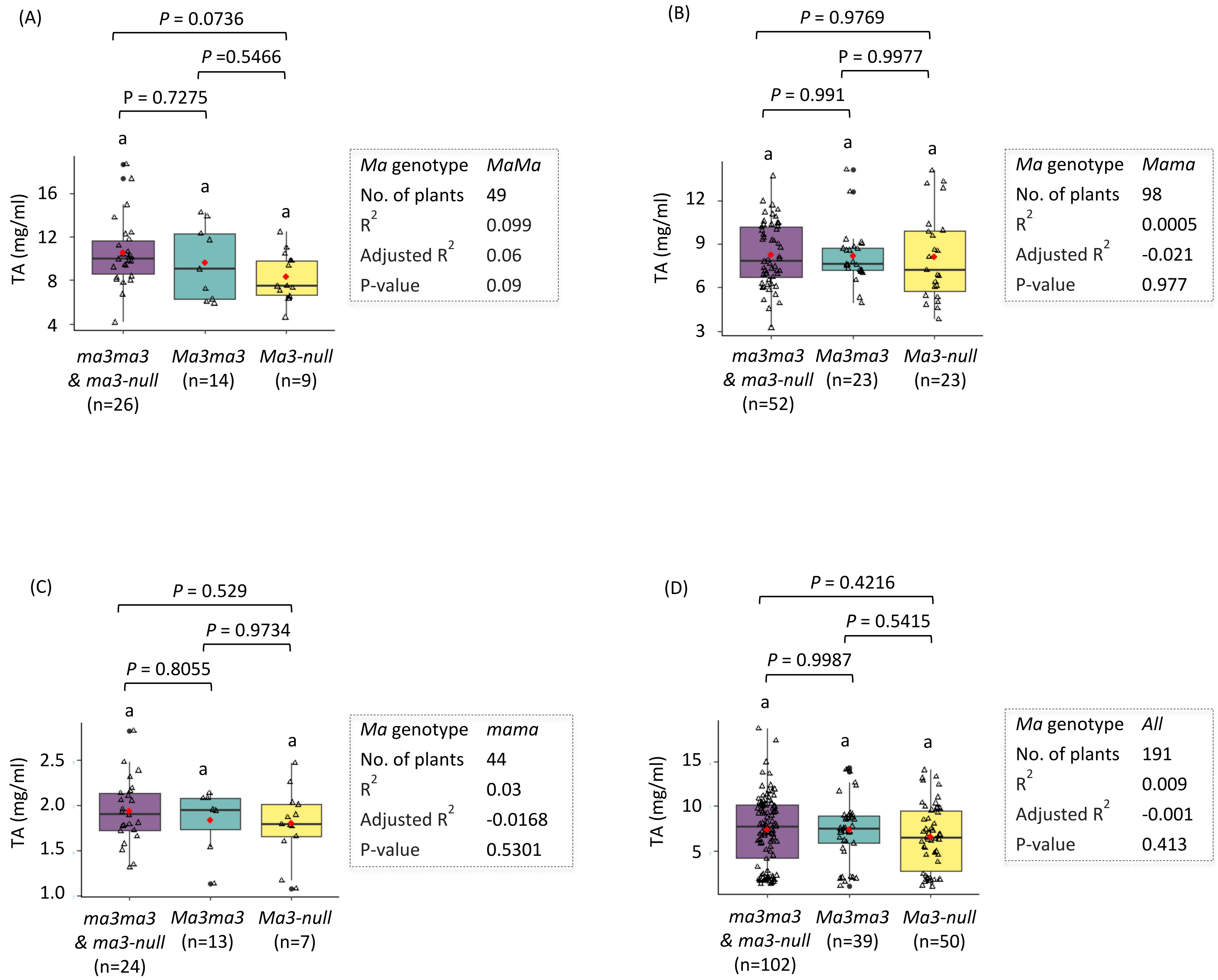
(A)
P = 0.0736
P =0.5466
P = 0.7275
a
a
a
TA (mg/ml)
16
12
8
4
ma3ma3 & ma3-null (n=26)
Ma3ma3 (n=14)
Ma3-null (n=9)
Ma genotype MaMa
No. of plants 49
R2 0.099
Adjusted R2 0.06
P-value 0.09
(B)
P = 0.9769
P = 0.9977
P = 0.991
a
a
a
TA (mg/ml)
12
9
6
3
ma3ma3 & ma3-null (n=52)
Ma3ma3 (n=23)
Ma3-null (n=23)
Ma genotype Mama
No. of plants 98
R2 0.0005
Adjusted R2 -0.021
P-value 0.977
(C)
P = 0.529
P = 0.9734
P = 0.8055
a
a
a
TA (mg/ml)
2.5
2.0
1.5
1.0
ma3ma3 & ma3-null (n=24)
Ma3ma3 (n=13)
Ma3-null (n=7)
Ma genotype mama
No. of plants 44
R2 0.03
Adjusted R2 -0.0168
P-value 0.5301
(D)
P = 0.4216
P = 0.5415
P = 0.9987
a
a
a
TA (mg/ml)
15
10
5
ma3ma3 & ma3-null (n=102)
Ma3ma3 (n=39)
Ma3-null (n=50)
Ma genotype All
No. of plants 191
R2 0.009
Adjusted R2 -0.001
P-value 0.413

Fig. S2

(A)

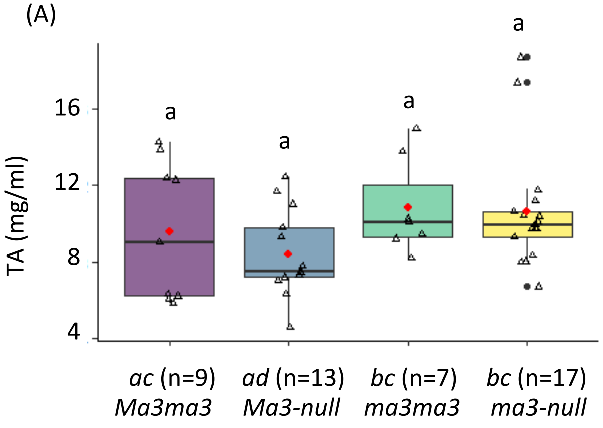

Ma genotype	*MaMa*
No. of plants	46
R^2	0.11
Adjusted R^2	0.046
P-value	0.1762

(B)

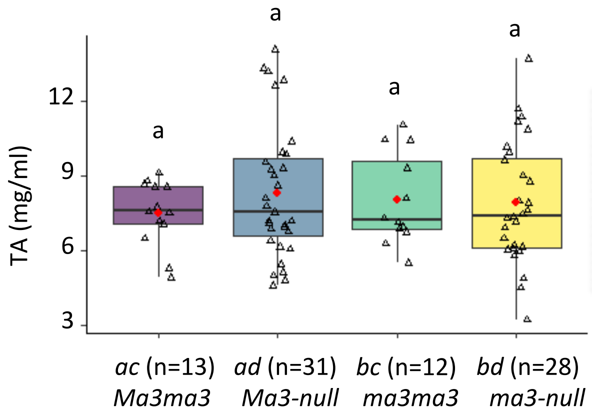

Ma genotype	*Mama*
No. of plants	84
R^2	0.015
Adjusted R^2	-0.022
P-value	0.744

(C)

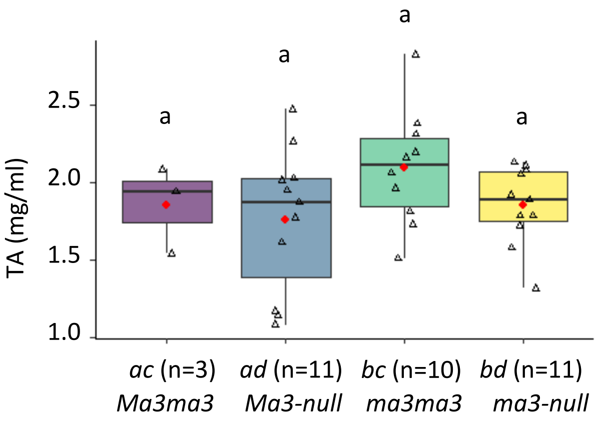

Ma genotype	*mama*
No. of plants	35
R^2	0.129
Adjusted R^2	0.045
P-value	0.2268

(D)

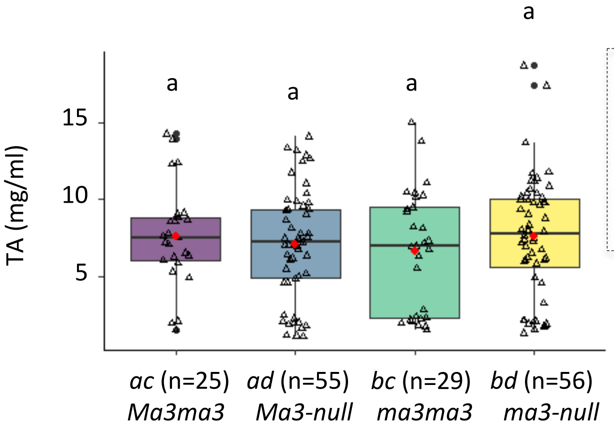

Ma genotype	*All*
No. of plants	165
R^2	0.009
Adjusted R^2	-0.009
P-value	0.6898

Fig. S3

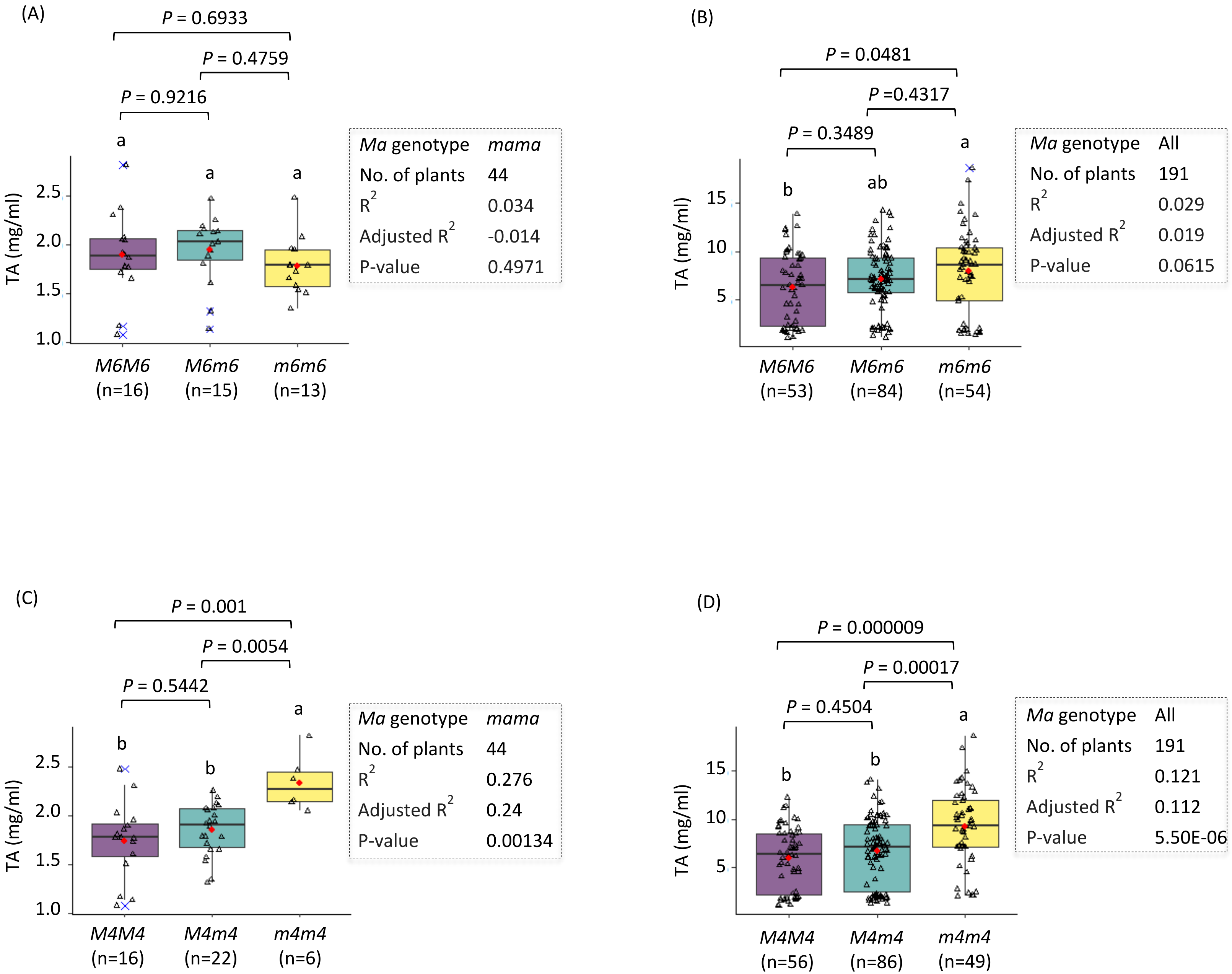
(A)
P = 0.6933
P = 0.4759
P = 0.9216
a
a
a
TA (mg/ml)
2.5
2.0
1.5
1.0
M6M6 (n=16)
M6m6 (n=15)
m6m6 (n=13)
Ma genotype mama
No. of plants 44
R2 0.034
Adjusted R2 -0.014
P-value 0.4971
(B)
P = 0.0481
P =0.4317
P = 0.3489
b
ab
a
TA (mg/ml)
15
10
5
M6M6 (n=53)
M6m6 (n=84)
m6m6 (n=54)
Ma genotype All
No. of plants 191
R2 0.029
Adjusted R2 0.019
P-value 0.0615
(C)
P = 0.001
P = 0.0054
P = 0.5442
b
b
a
TA (mg/ml)
2.5
2.0
1.5
1.0
M4M4 (n=16)
M4m4 (n=22)
m4m4 (n=6)
Ma genotype mama
No. of plants 44
R2 0.276
Adjusted R2 0.24
P-value 0.00134
(D)
P = 0.000009
P = 0.00017
P = 0.4504
b
b
a
TA (mg/ml)
15
10
5
M4M4 (n=56)
M4m4 (n=86)
m4m4 (n=49)
Ma genotype All
No. of plants 191
R2 0.121
Adjusted R2 0.112
P-value 5.50E-06

Fig. S4

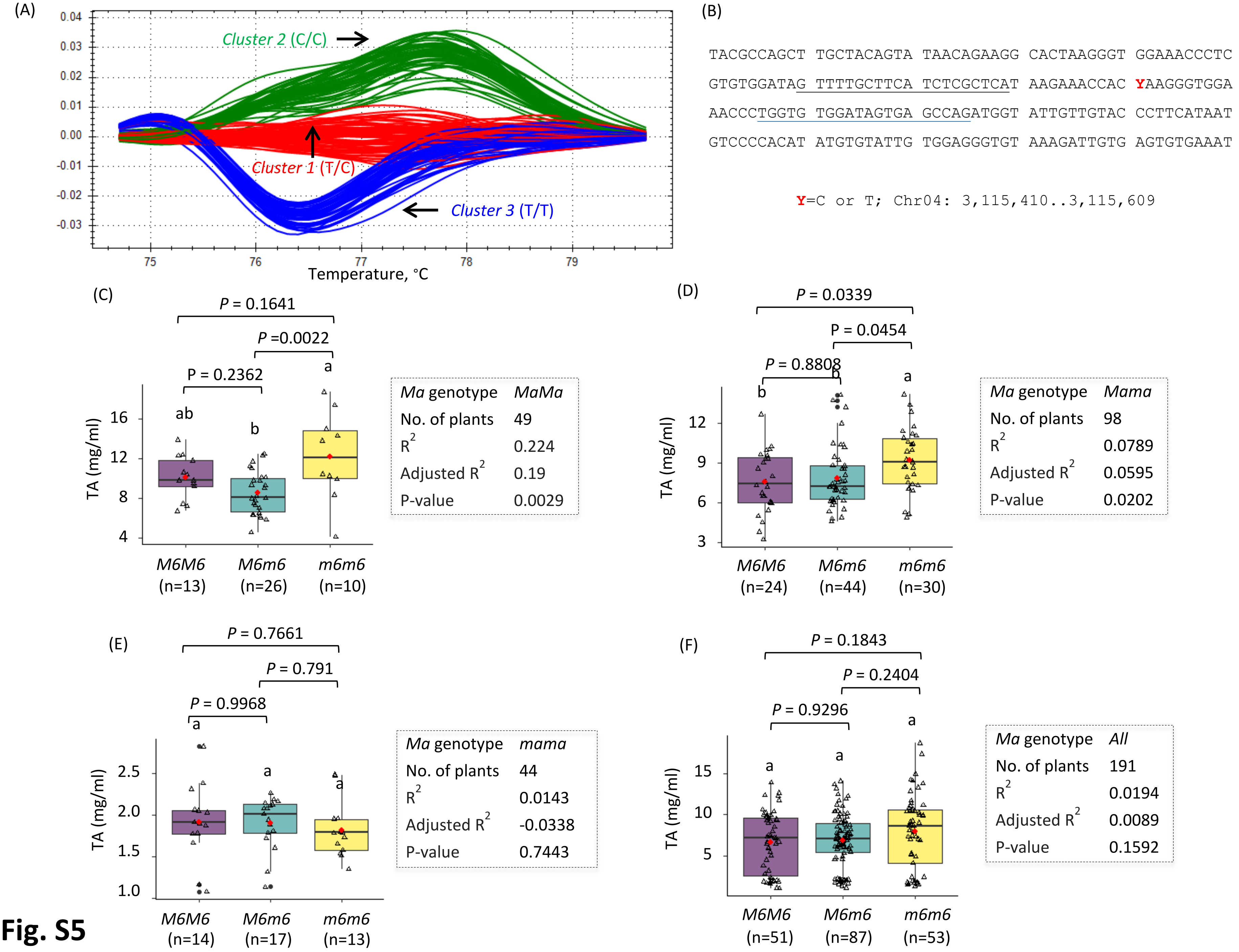

(A)
Cluster 2 (C/C)
Cluster 1 (T/C)
Cluster 3 (T/T)
Temperature, °C
(B)
TACGCCAGCT TGCTACAGTA TAACAGAAGG CACTAAGGGT GGAAACCCTC
GTGTGGATAG TTTTGCTTCA TCTCGCTCAT AAGAAACCAC YAAGGGTGGA
AACCCTGGTG TGGATAGTGA GCCAGATGGT ATTGTTGTAC CCTTCATAAT
GTCCCCACAT ATGTGTATTG TGGAGGGTGT AAAGATTGTG AGTGTGAAAT
Y=C or T; Chr04: 3,115,410..3,115,609
(C)
P = 0.1641
P =0.0022
P = 0.2362
TA (mg/ml)
M6M6 (n=13)
M6m6 (n=26)
m6m6 (n=10)
Ma genotype MaMa
No. of plants 49
R2 0.224
Adjusted R2 0.19
P-value 0.0029
(D)
P = 0.0339
P = 0.0454
P = 0.8808
TA (mg/ml)
M6M6 (n=24)
M6m6 (n=44)
m6m6 (n=30)
Ma genotype Mama
No. of plants 98
R2 0.0789
Adjusted R2 0.0595
P-value 0.0202
(E)
P = 0.7661
P = 0.791
P = 0.9968
TA (mg/ml)
M6M6 (n=14)
M6m6 (n=17)
m6m6 (n=13)
Ma genotype mama
No. of plants 44
R2 0.0143
Adjusted R2 -0.0338
P-value 0.7443
(F)
P = 0.1843
P = 0.2404
P = 0.9296
TA (mg/ml)
M6M6 (n=51)
M6m6 (n=87)
m6m6 (n=53)
Ma genotype All
No. of plants 191
R2 0.0194
Adjusted R2 0.0089
P-value 0.1592

Fig. S5

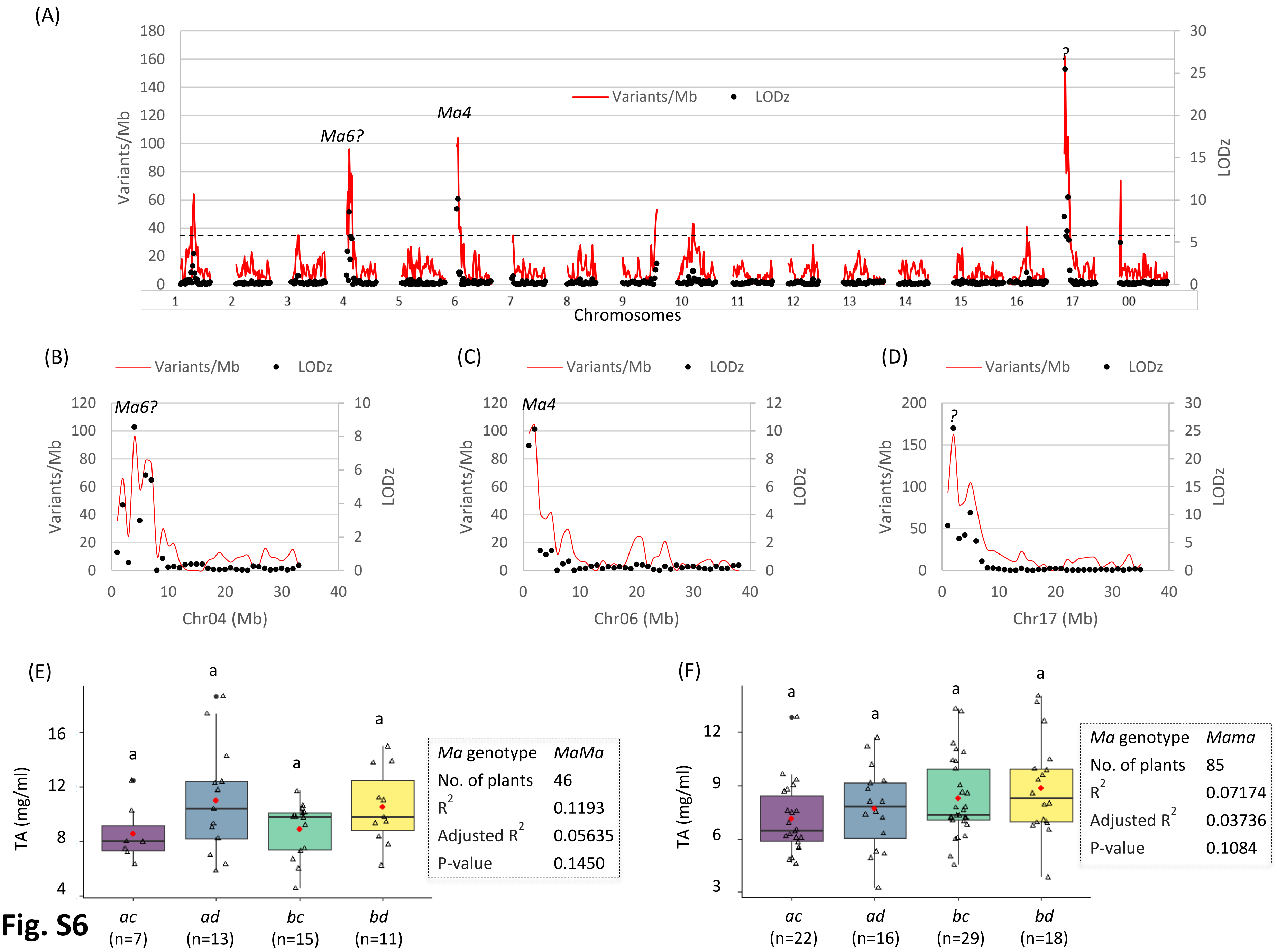
(A)
Variants/Mb
LODz
Ma6?
Ma4
?
Chromosomes
1 2 3 4 5 6 7 8 9 10 11 12 13 14 15 16 17 00
(B)
Ma6?
Chr04 (Mb)
(C)
Ma4
Chr06 (Mb)
(D)
?
Chr17 (Mb)
(E)
TA (mg/ml)
ac (n=7)
ad (n=13)
bc (n=15)
bd (n=11)
Ma genotype MaMa
No. of plants 46
R2 0.1193
Adjusted R2 0.05635
P-value 0.1450
(F)
TA (mg/ml)
ac (n=22)
ad (n=16)
bc (n=29)
bd (n=18)
Ma genotype Mama
No. of plants 85
R2 0.07174
Adjusted R2 0.03736
P-value 0.1084

Fig. S6

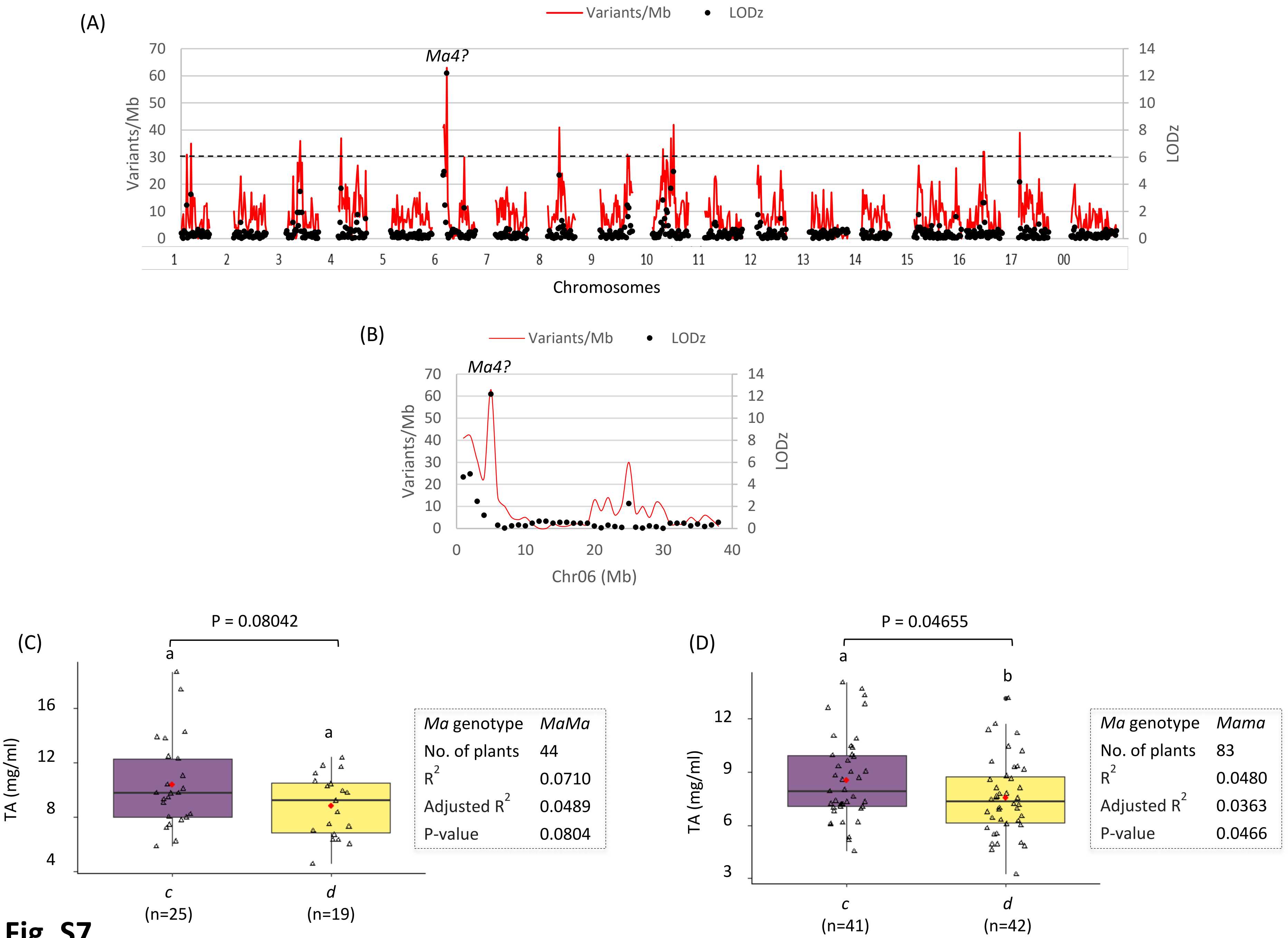
(A)
Variants/Mb
LODz
Ma4?
Variants/Mb
LODz
Chromosomes
1 2 3 4 5 6 7 8 9 10 11 12 13 14 15 16 17 00
(B)
Variants/Mb
LODz
Ma4?
Variants/Mb
LODz
Chr06 (Mb)
(C)
P = 0.08042
a
a
TA (mg/ml)
c
(n=25)
d
(n=19)
Ma genotype MaMa
No. of plants 44
R2 0.0710
Adjusted R2 0.0489
P-value 0.0804
(D)
P = 0.04655
a
b
TA (mg/ml)
c
(n=41)
d
(n=42)
Ma genotype Mama
No. of plants 83
R2 0.0480
Adjusted R2 0.0363
P-value 0.0466

Fig. S7

Table S1. Primer sequences of DNA markers

Marker name	Primer Name	Forward primer sequence (5' to 3')	Reverse primer sequence (5' to 3')	Notes
HRM-Ma6	Chr04_08022HF/R	CACCAATCCTTCACAACCA	ACAGATGTTTGTTTGGATGA	Targeting base 8,022,967 on chromosome 4 for mapping confirmation of Ma6
SSR_Ma6	SSR_Ch04_08133_F/R	AATAAAACGGTCCCGAATCC	AAACTCTGAAAGCGGACGAA	Detecting an SSR region located at the 8133th kb on chromosome 4.
HRM2-Ma6	Chr4_03115_F/R	GTTTTGCTTCATCTCGCTCA	CTGGCTCACTATCCACACCA	Targeting base 3115500 on chromosome 4 for examining the independence of an SNV peak 4-5 Mb upstream of Ma6.
HRM-Ma4	Ch6_01614HF/R	AACATGATGGTTTCAGGGGTA	GGACATATTTATGAGATTTTTGAACC	Targeting base 1,614,796 on chromosome 6 for mapping confirmation of Ma4
SSR_Ma4	SSR_ Chr6_01494F/R	CCGCCTTTCTATTCAAACCA	TGGGTTTTCCTTGTGCATTT	Detecting an SSR region located at the 1494th kb on chromosome 6.
C14087	C14087F/R	CACCGCGTCAAAAATACCTT	CTTGTTGTTTCCCTCCCAAA	Detecting an SSR region located at the 4908th kb on chromosome 6. Wang et al (2012)
C1902	C1902F/R	CCTCTGCCTCTCCCAAAATA	ATCGTCGTGACCAGAAGGAC	Detecting an SSR region located at the 1540th kb on chromosome 17. Wang et al (2012)
CAPS1455	CAPS1455_F/R	GCCGCTTCTGGACTATCACTA	TTCTTCAACCGCAAACTCCT	Ma genotyping (Bai et al 2012)
CH02g09	CH02g09F/R	TCAGACAGAAGAGGAACTGTATTTG	CAAACAAACCAGTACCGCAA	Detecting an SSR region located at the 10,000th kb on chromosome 8. Wang et al (2012)
MdPP2CH	MdPP2CH-F/R	TGATTCCCTCTTGGATGTTGG	CTCCTTTTCCGTGCCTCTTTG	Ma3 genotyping (Jia et al 2018)
MdSAUR37	MdSAUR37F/R	CTACAATCGGAGACAATGACAGAG	GAGCAAGTTCAAGTTGATTTAC	Ma3 genotyping (Jia et al 2018)

Table S2. F_1 segregants used for pooling of genomes and their acidity and genotypes at loci *Ma (CAPS1455)* and *Ma3 (MdPP2CH and MdSAUR37)*

Tree #	Malic acid (g/l)	Ma (CAPS1455)[a]	Ma3 (MdPP2CH)[b]	Ma3 (MdSAUR37)[b]	Genome pool
6_65	12.3	MaMa	Ma3ma3	Ma3ma3	High acidity
7_60	12.6	Mama	Ma3ma3	Ma3ma3	High acidity
7_76	11.0	MaMa	Ma3ma3	Ma3Ma3	High acidity
6_103	14.1	Mama	Ma3ma3	Ma3Ma3	High acidity
6_153	10.4	Mama	Ma3ma3	Ma3Ma3	High acidity
6_87	9.6	Mama	Ma3ma3	Ma3Ma3	High acidity
7_39	12.9	Mama	Ma3ma3	Ma3Ma3	High acidity
7_7	13.3	Mama	Ma3ma3	Ma3Ma3	High acidity
6_84	15.0	MaMa	Ma3ma3	ma3ma3	High acidity
6_92	17.4	MaMa	Ma3ma3	ma3ma3	High acidity
7_15	18.7	MaMa	Ma3ma3	ma3ma3	High acidity
6_168	10.9	Mama	Ma3ma3	ma3ma3	High acidity
6_74	11.2	Mama	Ma3ma3	ma3ma3	High acidity
6_75	10.5	Mama	Ma3ma3	ma3ma3	High acidity
6_95	11.4	Mama	Ma3ma3	ma3ma3	High acidity
7_4	10.4	Mama	Ma3ma3	ma3ma3	High acidity
7_48	13.7	Mama	Ma3ma3	ma3ma3	High acidity
7_63	11.1	Mama	Ma3ma3	ma3ma3	High acidity
7_25	6.0	MaMa	Ma3ma3	Ma3ma3	Regular acidity
7_8	5.9	MaMa	Ma3ma3	Ma3ma3	Regular acidity
7_65	6.3	MaMa	Ma3ma3	Ma3Ma3	Regular acidity
7_71	6.3	MaMa	Ma3ma3	Ma3Ma3	Regular acidity
6_137	4.6	Mama	Ma3ma3	Ma3Ma3	Regular acidity
6_94	5.5	Mama	Ma3ma3	Ma3Ma3	Regular acidity
7_21	4.8	Mama	Ma3ma3	Ma3Ma3	Regular acidity
7_36	6.1	Mama	Ma3ma3	Ma3Ma3	Regular acidity
7_72	5.0	Mama	Ma3ma3	Ma3Ma3	Regular acidity
6_140	6.7	MaMa	Ma3ma3	ma3ma3	Regular acidity
6_105	6.3	Mama	Ma3ma3	ma3ma3	Regular acidity
6_116	6.0	Mama	Ma3ma3	ma3ma3	Regular acidity
6_86	6.2	Mama	Ma3ma3	ma3ma3	Regular acidity
7_32	4.9	Mama	Ma3ma3	ma3ma3	Regular acidity
7_34	6.1	Mama	Ma3ma3	ma3ma3	Regular acidity
7_40	5.5	Mama	Ma3ma3	ma3ma3	Regular acidity
7_45	4.5	Mama	Ma3ma3	ma3ma3	Regular acidity
7_52	6.2	Mama	Ma3ma3	ma3ma3	Regular acidity
7_59	5.8	Mama	Ma3ma3	ma3ma3	Regular acidity
7_73	6.1	Mama	Ma3ma3	ma3ma3	Regular acidity

[a] Bai et al, 2012

Table S3. Comparison in the progeny of GMAL 4595 that were genotyped by both MdSAUR37 and CH02g09

	CH02g09 (ab x c-null or Ma3ma3 x ma3-null)				
MdSAUR37 (ab x b-null or Ma3ma3 X ma3-null)	ac (Ma3ma3)	ad (Ma3-null)	bc (ma3ma3)	bd (ma3-null)	sum
bb & b-null (ma3ma3 & ma3-null)	1	6	**28**	**53**	88
a-null (Ma3-null)	1	**39**	0	2	42
ab (Ma3ma3)	**23**	10	1	1	35
Sum	25	55	29	56	165

Note: The segregation at locus MdSAUR37 was normal for both parents. However, the segregation at locus CH02g09 for pollen parent PI 613988 was abnormal (54:111) although the segregation for Gala was normal (80:85). Viewing the linkage map of chromosome 8 constructed for PI 613988 (Wang et al 2012), all SSR markers from the chromosome proximal end through marker CH02g09 were highly distorted. However, the other markers following it had a normal or slightly distorted segregation. This suggested that the chromosomal segments housing markers CH02g09 and MdSAUR37 could segregate differently, which would explain why the segregation was distorted for CH02g09 while normal for MdSAUR37. A possible mechanism would be that pollens were more viable in those carrying the chromosomal segment with the null allele of CH02g09 than those without it. The use of marker CH02g09 was necessary as it could unequivocally genotype the progeny into all four possible genotypes of *Ma3*, which was essential to estimate the effect of *Ma3*. In contrast, genotypes *ma3ma3* (low acidity genotype) and *ma3-null* were indistinguishable to marker MdSAUR37, making it not ideal, if not unfit, to evaluate the effect of *Ma3*.

Table S4. Summary of reads mapping in pooled genome sequencing analysis

Pools	Reads category	Count	Percentage of reads	Average length	Number of bases	Percentage of bases	Coverage
High acidity pool	References	18	-	39,420,077.28	709,561,391	-	
	Mapped reads	110,682,421	70.35%	129.03	14,281,644,116	73.47%	20.1
	Not mapped reads	46,646,669	29.65%	110.54	5,156,416,463	26.53%	7.3
	Reads in pairs	79,151,476	50.31%	422.28	10,661,195,325	54.85%	15
	Broken paired reads	31,530,945	20.04%	114.82	3,620,448,791	18.63%	5.1
	Total reads	157,329,090	100.00%	123.55	19,438,060,579	100.00%	27.4
Regular acidity pool	Mapped reads	163,005,544	72.47%	130.82	21,324,149,210	74.70%	30.1
	Not mapped reads	61,923,556	27.53%	116.61	7,221,015,171	25.30%	10.2
	Reads in pairs	118,404,708	52.64%	425.17	15,996,623,071	56.04%	22.5
	Broken paired reads	44,600,836	19.83%	119.45	5,327,526,139	18.66%	7.5
	Total reads	224,929,100	100.00%	126.91	28,545,164,381	100.00%	40.2

Table S5. List of all genes annotated under the peak of QTL Ma6 in the apple reference genome (Daccord et al 2017)

Name	Chromosome	Region	Annotation by MAPMAN	Annotation description
MD04G1060800	Chr04	8013339..8017031	M35.1 // not assigned.no ontology	2-oxoglutarate (2OG) and Fe(II)-dependent oxygenase superfamily protein
MD04G1060900	Chr04	8017657..8024612	M29.5.11.4.2 // protein.degradation.ubiquitin.E3.RING	ubiquitin-protein ligases
MD04G1061000	Chr04	8046050..8047641	M35.2 // not assigned.unknown	Protein of unknown function (DUF1639)
MD04G1061100	Chr04	8055753..8055867		5S_rRNA
MD04G1061200	Chr04	8078422..8080537	M27.3.22 // RNA.regulation of transcription.HB,Homeobox transcription factor family	homeobox protein 2
MD04G1061300	Chr04	8083261..8085009	M10.5.4 // cell wall.cell wall proteins.HRGP	hydroxyproline-rich glycoprotein family protein
MD04G1061400	Chr04	8091715..8094707	M35.1.5 // not assigned.no ontology.pentatricopeptide (PPR) repeat-containing protein	Tetratricopeptide repeat (TPR)-like superfamily protein
MD04G1061500	Chr04	8132582..8135995	M35.2 // not assigned.unknown	Phosphotyrosine protein phosphatases superfamily protein
MD04G1061600	Chr04	8136590..8141994	M11.9.1 // lipid metabolism.lipid degradation.palmitoyl[protein] hydrolase	alpha/beta-Hydrolases superfamily protein
MD04G1061700	Chr04	8152588..8153553	M29.4 // protein.posttranslational modification	Protein kinase superfamily protein
MD04G1061800	Chr04	8166360..8167298	M29.4 // protein.posttranslational modification	Protein kinase superfamily protein
MD04G1061900	Chr04	8167975..8175412	M27.3.67 // RNA.regulation of transcription.putative transcription regulator	a CCHC-type zinc finger
MD04G1062000	Chr04	8176477..8178741	M29.2.1.2.2.15 // protein.synthesis.ribosomal protein.eukaryotic.60S subunit.L15	Ribosomal protein L23/L15e family protein
MD04G1062100	Chr04	8179353..8180114	M16.5.1.1.1.4 // secondary metabolism.sulfur-containing.glucosinolates.synthesis.aliphatic.methylthioalkylmalate isomerase small subunit (MAM-IS)	Aconitase/3-isopropylmalate dehydratase protein
MD04G1062200	Chr04	8182369..8199514	M28.2 // DNA.repair	MUTS-like protein 4
MD04G1062300	Chr04	8203804..8205305	M35.2 // not assigned.unknown	No annotation
MD04G1062400	Chr04	8206702..8211760	M35.1 // not assigned.no ontology	Oxidoreductase family protein
MD04G1062500	Chr04	8215031..8216039	M35.2 // not assigned.unknown	Protein of unknown function
MD04G1062600	Chr04	8226122..8230614	M23.4.3 // nucleotide metabolism.phosphotransfer and pyrophosphatases.uridylate kinase	P-loop containing nucleoside triphosphate hydrolases superfamily protein
MD04G1062700	Chr04	8231269..8234696	M27.3.67 // RNA.regulation of transcription.putative transcription regulator	Methyltransferase MT-A70 family protein
MD04G1062800	Chr04	8260047..8273261	M35.1.2 // not assigned.no ontology.agenet domain-containing protein	G2484-1 protein
MD04G1062900	Chr04	8288449..8293424	M35.2 // not assigned.unknown	O-fucosyltransferase family protein
MD04G1063000	Chr04	8325353..8326444	M29.5.7 // protein.degradation.metalloprotease	Matrixin family protein
MD04G1063100	Chr04	8328944..8331113	M35.2 // not assigned.unknown	Protein of unknown function
MD04G1063200	Chr04	8364419..8364754	M16.8.2 // secondary metabolism.flavonoids.chalcones	NAD(P)-linked oxidoreductase superfamily protein
MD04G1063300	Chr04	8373208..8377275	M33.99 // development.unspecified	Nodulin MtN21 /EamA-like transporter family protein
MD04G1063400	Chr04	8379956..8381112	M29.4 // protein.posttranslational modification	Protein kinase superfamily protein
MD04G1063500	Chr04	8386924..8388495	M29.4 // protein.posttranslational modification	Protein kinase superfamily protein

MD04G1063600	Chr04	8409858..8410856	M27.3.11 // RNA.regulation of transcription.C2H2 zinc finger family	C2H2-like zinc finger protein
MD04G1063700	Chr04	8416801..8418219	M23 // nucleotide metabolism	histidine triad nucleotide-binding 4
MD04G1063800	Chr04	8432676..8437714	M26.6 // misc.O-methyl transferases	protein arginine methyltransferase 7
MD04G1063900	Chr04	8491238..8492064	M35.2 // not assigned.unknown	No annotation
MD04G1064000	Chr04	8505855..8512785	M35.2 // not assigned.unknown	No annotation
MD04G1064100	Chr04	8566578..8566805	M20.2 // stress.abiotic	osmotin 34
MD04G1064200	Chr04	8643210..8643887	M20.2 // stress.abiotic	osmotin 34
MD04G1064300	Chr04	8670563..8672537	M35.2 // not assigned.unknown	No annotation
MD04G1064400	Chr04	8673633..8674310	M20.2 // stress.abiotic	osmotin 34
MD04G1064500	Chr04	8683679..8686436	M27.3.25 // RNA.regulation of transcription.MYB domain transcription factor family	myb domain protein 3r-4
MD04G1064600	Chr04	8687642..8691096	M10.1.2 // cell wall.precursor synthesis.UGE	UDP-D-glucose/UDP-D-galactose 4-epimerase 1
MD04G1064700	Chr04	8697125..8698967	M20.2.1 // stress.abiotic.heat /// M27.3.23 // RNA.regulation of transcription.HSF,Heat-shock transcription factor family	winged-helix DNA-binding transcription factor family protein
MD04G1064800	Chr04	8714058..8714648	M35.2 // not assigned.unknown	BON association protein 2
MD04G1064900	Chr04	8716704..8719942	M29.5.11.4.2 // protein.degradation.ubiquitin.E3.RING	C3HC4 type (RING finger) family protein
MD04G1065000	Chr04	8734043..8738403	M35.2 // not assigned.unknown	No annotation
MD04G1065100	Chr04	8741662..8744334	M35.2 // not assigned.unknown	No annotation
MD04G1065200	Chr04	8753782..8757499	M28.1 // DNA.synthesis/chromatin structure	replication factor C 2
MD04G1065300	Chr04	8758017..8759740	M29.2.1.2.2.24 // protein.synthesis.ribosomal protein.eukaryotic.60S subunit.L24	ribosomal protein L24
MD04G1065400	Chr04	8763878..8763989		5S_rRNA
MD04G1065500	Chr04	8771410..8774548	M29.5.5 // protein.degradation.serine protease	RHOMBOID-like 2
MD04G1065600	Chr04	8782065..8787149	M30.3 // signalling.calcium	IQ-domain 22
MD04G1065700	Chr04	8790230..8790341		5S_rRNA
MD04G1065800	Chr04	8790596..8790703		5S_rRNA
MD04G1065900	Chr04	8792343..8792426		tRNA-Pseudo
MD04G1066000	Chr04	8814106..8819506	M30.6 // signalling.MAP kinases	PAS domain-containing protein tyrosine kinase family protein
MD04G1066100	Chr04	8824435..8830841	M35.2 // not assigned.unknown	hapless 2
MD04G1066200	Chr04	8832781..8833902	M35.2 // not assigned.unknown	No annotation
MD04G1066300	Chr04	8844411..8847733	M26.10 // misc.cytochrome P450	polypeptide 2
MD04G1066400	Chr04	8931656..8934297	M26.10 // misc.cytochrome P450	polypeptide 2
MD04G1066500	Chr04	8964185..8970098	M31.2 // cell.division	GPI transamidase subunit PIG-U
MD04G1066600	Chr04	8981094..8985122	M35.2 // not assigned.unknown	No annotation
MD04G1066700	Chr04	8989440..8996174	M31.4 // cell.vesicle transport	Ubiquitin-like superfamily protein
MD04G1066800	Chr04	8999281..9002402	M27.3.21 // RNA.regulation of transcription.GRAS transcription factor family	GRAS family transcription factor

Table S6. List of all genes annotated under the peak of QTL *Ma4* in the apple reference genome (Daccord et al 2017)

Name	Chromosome	Region	Annotation by MAPMAN	Annotation description
MD06G1008900	Chr06	1013698..1014431	M20.2.3 // stress.abiotic.drought/salt	staurosporin and temperature sensitive 3-like b
MD06G1009000	Chr06	1028933..1032879	M21.6 // redox.dismutases and catalases	catalase 2
MD06G1009100	Chr06	1036360..1038508	M35.2 // not assigned.unknown	Other Eukaryotes-0 (source: NCBI BLink).
MD06G1009200	Chr06	1050052..1052738	M33.99 // development.unspecified	Plant regulator RWP-RK family protein
MD06G1009300	Chr06	1062065..1063007	M27.3.60 // RNA.regulation of transcription.NIN-like bZIP-related family	Plant regulator RWP-RK family protein
MD06G1009400	Chr06	1064533..1079801	M35.1 // not assigned.no ontology	Patched family protein
MD06G1009500	Chr06	1090086..1090202		5S_rRNA
MD06G1009600	Chr06	1097251..1105395	M29.4.1.57 // protein.posttranslational modification.kinase.receptor like cytoplasmatic kinase VII	Protein kinase superfamily protein
MD06G1009700	Chr06	1117871..1121912	M29.4.1.57 // protein.posttranslational modification.kinase.receptor like cytoplasmatic kinase VII	Protein kinase superfamily protein
MD06G1009800	Chr06	1166275..1168152	M35.2 // not assigned.unknown	Domain of unknown function (DUF23)
MD06G1009900	Chr06	1169763..1169864		No annotation
MD06G1010000	Chr06	1170406..1174932	M27.3.42 // RNA.regulation of transcription.Bromodomain proteins	DNA-binding bromodomain-containing protein
MD06G1010100	Chr06	1183409..1185741	M35.2 // not assigned.unknown	No annotation
MD06G1010200	Chr06	1205193..1206907	M35.2 // not assigned.unknown	No annotation
MD06G1010300	Chr06	1210398..1211868	M35.2 // not assigned.unknown	Protein of unknown function (DUF833)
MD06G1010400	Chr06	1214169..1221898	M28.99 // DNA.unspecified	FAR1-related sequence 3
MD06G1010500	Chr06	1242533..1242969	M35.2 // not assigned.unknown	No annotation
MD06G1010600	Chr06	1244001..1244396	M35.2 // not assigned.unknown	Plant self-incompatibility protein S1 family
MD06G1010700	Chr06	1252243..1252862		No annotation
MD06G1010800	Chr06	1258804..1261663	M18 // Co-factor and vitamine metabolism	Biotin/lipoate A/B protein ligase family
MD06G1010900	Chr06	1314301..1317373	M17.1.1.2.1 // hormone metabolism.abscisic acid.synthesis-degradation.degradation.8-hydroxylase /// M26.10 // misc.cytochrome P450	polypeptide 3
MD06G1011000	Chr06	1329502..1331401	M35.2 // not assigned.unknown	No annotation
MD06G1011100	Chr06	1332079..1336111	M29.2.1.1.1.2.22 // protein.synthesis.ribosomal protein.prokaryotic.chloroplast.50S subunit.L22	Ribosomal protein L22p/L17e family protein
MD06G1011200	Chr06	1336895..1343056	M35.2 // not assigned.unknown	decapping 5-like
MD06G1011300	Chr06	1343060..1345538	M35.2 // not assigned.unknown	Protein of unknown function (DUF1218)
MD06G1011400	Chr06	1376336..1379040	M35.1 // not assigned.no ontology	Uncharacterised protein family (UPF0497)
MD06G1011500	Chr06	1383072..1383296	M3.1.1.1 // minor CHO metabolism.raffinose family.galactinol synthases.known /// M3.1.1.2 // minor CHO metabolism.raffinose family.galactinol synthases.putative	galactinol synthase 1
MD06G1011600	Chr06	1463717..1465165	M35.1.5 // not assigned.no ontology.pentatricopeptide (PPR) repeat-containing protein	Tetratricopeptide repeat (TPR)-like superfamily protein
MD06G1011700	Chr06	1468343..1476149	M33.99 // development.unspecified	embryo sac development arrest 7
MD06G1011800	Chr06	1481594..1481730		MIR168

MD06G1011900	Chr06	1490828..1494993	M35.2 // not assigned.unknown	Protein of unknown function
MD06G1012000	Chr06	1504561..1505175	M35.2 // not assigned.unknown	Protein of unknown function
MD06G1012100	Chr06	1506508..1511063	M35.1 // not assigned.no ontology	RNA-binding (RRM/RBD/RNP motifs) family protein
MD06G1012200	Chr06	1531953..1534667	M26.9 // misc.glutathione S transferases	Glutathione S-transferase family protein
MD06G1012300	Chr06	1534669..1537367	M30.5 // signalling.G-proteins	RHO guanyl-nucleotide exchange factor 14
MD06G1012400	Chr06	1585017..1586498	M35.2 // not assigned.unknown	ribosomal protein L16
MD06G1012500	Chr06	1590799..1599810	M27.3.57 // RNA.regulation of transcription.JUMONJI family /// M29.5.11.4.2 // protein.degradation.ubiquitin.E3.RING	transcription factor jumonji (jmjC) domain-containing protein
MD06G1012600	Chr06	1608429..1615091	M2.2.2.1.2 // major CHO metabolism.degradation.starch.starch cleavage.beta amylase	beta-amylase 2
MD06G1012700	Chr06	1630480..1630600		5S_rRNA
MD06G1012800	Chr06	1630844..1630955		5S_rRNA
MD06G1012900	Chr06	1634018..1639343	M10.8.2 // cell wall.pectin*esterases.acetyl esterase	Pectinacetylesterase family protein
MD06G1013000	Chr06	1642935..1651318	M29.5.11.4.2 // protein.degradation.ubiquitin.E3.RING	RING/U-box superfamily protein
MD06G1013100	Chr06	1660912..1661163	M27.3.24 // RNA.regulation of transcription.MADS box transcription factor family	MADS-box transcription factor family protein
MD06G1013200	Chr06	1676770..1677483	M27.3.24 // RNA.regulation of transcription.MADS box transcription factor family	AGAMOUS-like 80
MD06G1013300	Chr06	1680972..1681502	M35.2 // not assigned.unknown	No annotation
MD06G1013400	Chr06	1684528..1685461		No annotation
MD06G1013500	Chr06	1694457..1694954	M27.3.24 // RNA.regulation of transcription.MADS box transcription factor family	AGAMOUS-like 80
MD06G1013600	Chr06	1720426..1721037	M27.3.24 // RNA.regulation of transcription.MADS box transcription factor family	AGAMOUS-like 80
MD06G1013700	Chr06	1725560..1739393	M12.4 // N-metabolism.misc	Aldolase-type TIM barrel family protein
MD06G1013800	Chr06	1741186..1741741	M29.2.4 // protein.synthesis.elongation	Translation elongationfactor EF1B/ribosomal protein S6 family protein
MD06G1013900	Chr06	1745452..1746342	M35.2 // not assigned.unknown	UDP-glucosyl transferase 73C7
MD06G1014000	Chr06	1746751..1746933	M16.8.4 // secondary metabolism.flavonoids.flavonols /// M17.4.1 // hormone metabolism.cytokinin.synthesis-degradation /// M20.1 // stress.biotic /// M26.2 // misc.UDP glucosyl and glucoronyl transferases	UDP-Glycosyltransferase superfamily protein
MD06G1014100	Chr06	1748981..1762210	M20.1 // stress.biotic	ENHANCED DISEASE RESISTANCE 2
MD06G1014200	Chr06	1803847..1807377	M35.2 // not assigned.unknown	Protein of unknown function
MD06G1014300	Chr06	1807691..1814526	M27.3.99 // RNA.regulation of transcription.unclassified	Mob1/phocein family protein
MD06G1014400	Chr06	1829232..1835824	M34.19.3 // transport.Major Intrinsic Proteins.NIP	DZC (Disease resistance/zinc finger/chromosome condensation-like region) domain containing protein
MD06G1014500	Chr06	1841069..1842458	M35.2 // not assigned.unknown	Protein of unknown function (DUF3464)
MD06G1014600	Chr06	1851659..1855037	M15 // metal handling	Putative membrane lipoprotein

MD06G1014700	Chr06	1876356..1881403	M27.3.50 // RNA.regulation of transcription.General Transcription /// M33.99 // development.unspecified	growth-regulating factor 5
MD06G1014800	Chr06	1885432..1889881	M29.4 // protein.posttranslational modification	Protein kinase superfamily protein
MD06G1014900	Chr06	1889883..1892152	M35.2 // not assigned.unknown	conserved peptide upstream open reading frame 25
MD06G1015000	Chr06	1894264..1899615	M20.2.3 // stress.abiotic.drought/salt	S-adenosyl-L-methionine-dependent methyltransferases superfamily protein
MD06G1015100	Chr06	1922680..1923164	M35.2 // not assigned.unknown	No annotation
MD06G1015200	Chr06	1938586..1942209	M28.1 // DNA.synthesis/chromatin structure	Replication factor-A protein 1-related
MD06G1015300	Chr06	1942676..1948290	M35.2 // not assigned.unknown	Protein of unknown function
MD06G1015400	Chr06	1948607..1955229	M35.1 // not assigned.no ontology	RINT-1 / TIP-1 family
MD06G1015500	Chr06	1956127..1958223	M35.1 // not assigned.no ontology	G10 family protein
MD06G1015600	Chr06	1959232..1964120	M35.2 // not assigned.unknown	No annotation
MD06G1015700	Chr06	1964269..1971138	M29.2.1.1.3.1.5 // protein.synthesis.ribosomal protein.prokaryotic.unknown organellar.30S subunit.S5	Ribosomal protein S5 family protein
MD06G1015800	Chr06	1985925..1987557		No annotation
MD06G1015900	Chr06	1987926..1990885	M35.2 // not assigned.unknown	No annotation
MD06G1016000	Chr06	1993249..1993671	M34.3 // transport.amino acids	Amino acid permease family protein
MD06G1016100	Chr06	1994036..2001939		No annotation
